# Salinity-driven microbial adaptation of hydrocarbon-degrading communities in coastal sediments

**DOI:** 10.1128/msphere.00369-26

**Published:** 2026-07-02

**Authors:** Yongyi Peng, Qing Liu, Xianbiao Lin, Fengmin Xing, Shengjie Li, Xinyue Liu, Yingchun Han, Yanbin Chen, Xiyang Dong

**Affiliations:** 1 Key Laboratory of Marine Genetic Resources, Third Institute of Oceanography, Ministry of Natural Resources 118477, Xiamen, China; 2 Department of Microbiology, Biomedicine Discovery Institute, Monash University 161661, Clayton, Victoria, Australia; 3 Frontiers Science Center for Deep Ocean Multispheres and Earth System, and Key Laboratory of Marine Chemistry Theory and Technology, Ministry of Education, Ocean University of China 825955, Qingdao, China; 4 Institute of Marine Biology and Pharmacology, Ocean College, Zhejiang University 601090, Zhoushan, China; 5 School of Oceanography, Shanghai Jiao Tong University 722366, Shanghai, China; Clemson University Department of Biological Sciences, Clemson, South Carolina, USA

**Keywords:** salinity gradient, hydrocarbon degradation, coastal microbiome, metagenomics, halotolerance, microbial adaptation

## Abstract

Salinity is a major abiotic driver of microbial diversity and metabolic function in coastal ecosystems. While its broad ecological impacts are well established, its role in shaping hydrocarbon-degrading communities and their adaptive mechanisms remains poorly understood. Here, we integrated gene- and genome-resolved metagenomics to investigate how salinity regulates the diversity, ecological interactions, and evolutionary dynamics of aerobic hydrocarbon-degrading microbes in Zhenhai Bay sediments (0.17–28.54 practical salinity units [PSU]). Across the natural salinity gradient, 10 types of hydrocarbon-degrading genes and 30 bacterial genomes spanning four phyla were identified, revealing extensive metabolic potential for the aerobic degradation of both aliphatic and aromatic hydrocarbons. The functional diversity and relative abundance of these genes increased significantly with salinity, accompanied by strong correlations with organic carbon parameters and nitrogen availability. Co-occurrence network analyses showed that hydrocarbon degraders, particularly *Gammaproteobacteria*, acted as key taxa maintaining community stability under saline conditions. Comparative genomics revealed that these bacteria possess multiple halotolerance strategies, including compatible solute biosynthesis and ion transport, supported by diverse energy-generating pathways. Frequent horizontal gene transfer and duplication of alkane monooxygenases (*alkB* and *cyp153*) expanded substrate ranges and enhanced functional diversity in hydrocarbon oxidation, highlighting salinity-driven evolutionary innovation. Together, these findings demonstrate that salinity governs the structure, metabolism, and evolution of hydrocarbon-degrading microbes, promoting microbial adaptation and functional diversification in coastal sediments.

**IMPORTANCE:**

Salinity is a defining feature of coastal ecosystems and a major regulator of microbial processes that support carbon cycling and pollutant degradation. This study highlights that salinity plays a central role in structuring hydrocarbon-degrading microbial communities and shaping their functional capacities and evolutionary trajectories in coastal sediments. By integrating osmoadaptation, metabolic potential, and community organization, our work shows that hydrocarbon degraders function as key links between environmental conditions and ecological processes. Salinity-driven shifts in microbial networks and metabolic strategies illustrate how environmental gradients can foster resilience and stability in highly dynamic coastal systems. Beyond advancing understanding of microbial responses, this study has potential implications for the rational design of bioremediation strategies targeting hydrocarbon pollutants in saline and estuarine environments.

## INTRODUCTION

Hydrocarbons, organic compounds composed solely of carbon and hydrogen atoms, are chemically inert and resistant to biological degradation due to their strong covalent bonds and hydrophobicity (1). In marine and coastal ecosystems, hydrocarbons are ubiquitous components of natural organic matter, originating from both geological seeps and biological production, and they also constitute a major source of reduced carbon fueling microbial metabolism (2–4). The microbial oxidation of hydrocarbons represents a fundamental process in the global carbon cycle, converting hydrophobic organic compounds into bioavailable intermediates and linking reduced carbon pools to the broader network of biogeochemical transformations (2, 5). Understanding how this process is regulated by environmental factors is therefore critical for elucidating microbial control of carbon turnover in dynamic marine sediments.

Aerobic hydrocarbon degradation, mediated by oxygenases such as alkane monooxygenases (*alkB*, *cyp153*, and *ladA*) and ring-hydroxylating dioxygenases (*ndo*, *tmo*, and *dmpO*), is the dominant pathway for hydrocarbon oxidation in aerobic environments (6–8). These enzymes catalyze the initial activation of aliphatic and aromatic hydrocarbons, generating intermediates that feed into central metabolic pathways such as the tricarboxylic acid cycle (9). Numerous phylogenetically diverse bacteria, including members of *Alcanivoraceae*, *Halieaceae*, *Marinobacteraceae*, and *Sphingomonadaceae*, possess these catabolic systems and contribute substantially to hydrocarbon transformation in marine sediments (10–12). Despite their widespread occurrence, the ecological and evolutionary processes structuring hydrocarbon-degrading microbial communities remain poorly understood, especially across environmental gradients where multiple selective pressures interact.

Among the various physicochemical factors that govern microbial metabolism in coastal systems, salinity exerts one of the most pervasive influences. Salinity shapes microbial community composition, enzyme function, and cell physiology by altering osmotic pressure, ion availability, and the energetic cost of maintaining homeostasis (11, 13, 14). Coastal and estuarine sediments often experience large fluctuations in salinity due to tidal exchange, freshwater inflow, and evaporation, resulting in strong spatial and temporal gradients. Such variation imposes selective constraints that may restructure microbial assemblages and favor the emergence of halotolerant lineages with specialized metabolic adaptations (15, 16). However, the mechanisms by which salinity modulates hydrocarbon-degrading communities and their functional gene repertoires remain largely unexplored. Existing studies report conflicting outcomes: some indicate that high salinity inhibits microbial activity and gene expression (17, 18), while others show that halotolerant taxa thrive under saline conditions and even expand their hydrocarbon-degradation potential (19). A comprehensive understanding of how salinity interacts with microbial metabolism, genetic diversity, and community assembly is therefore needed to elucidate the ecological principles underlying hydrocarbon turnover in variable coastal environments.

Here, we investigated the ecological and evolutionary determinants of aerobic hydrocarbon degradation along a pronounced salinity gradient in Zhenhai Bay, a semi-enclosed coastal ecosystem subject to strong tidal mixing and terrestrial inputs (0.17–28.54 PSU) (20). Using gene- and genome-resolved metagenomics, we characterized the taxonomic diversity, metabolic potential, and adaptive traits of hydrocarbon-degrading microbial communities across the gradient. Specifically, we examined how salinity influences the distribution of key hydrocarbon-degrading genes, shapes microbial network organization, and drives evolutionary processes such as horizontal gene transfer (HGT) and gene duplication. Our results reveal that salinity shapes the diversity, functional structure, and evolutionary dynamics of hydrocarbon-degrading microbes, providing new insights into how environmental gradients regulate carbon transformation in coastal sediments.

## RESULTS

### Salinity-driven assembly and diversity patterns of sediment microbiomes in Zhenhai Bay

Surface sediments were collected from 12 stations along Zhenhai Bay on the coast of the South China Sea during both winter and summer (Fig. S1). Physicochemical analyses revealed pronounced seasonal and spatial variability in key environmental variables, including temperature, inorganic nutrients, and pigments, with salinity showing the steepest gradient, ranging from 0.17 PSU at the upstream sites to 28.54 PSU near the estuarine mouth (Table S2). Based on these measurements, the sampling sites were grouped into three salinity regimes: low-, mid-, and high-salinity. Community profiling based on universal single-copy ribosomal protein genes showed that the sediment microbiomes were dominated by bacteria, primarily *Proteobacteria* (27.5% ± 1.4%), followed by *Chloroflexi* (8.0% ± 0.4%), *Desulfobacterota* (7.5% ± 0.6%), *Planctomycetota* (6.7% ± 0.2%), and *Acidobacteriota* (5.9% ± 0.2%; Table S3; Fig. S3). The predominance of *Proteobacteria* is consistent with global coastal-sediment trends (21, 22). Notably, their relative abundance increased steadily from low- to high-salinity sediments (26.1%–28.6%), indicating enrichment of halotolerant lineages.

**Fig 1:**
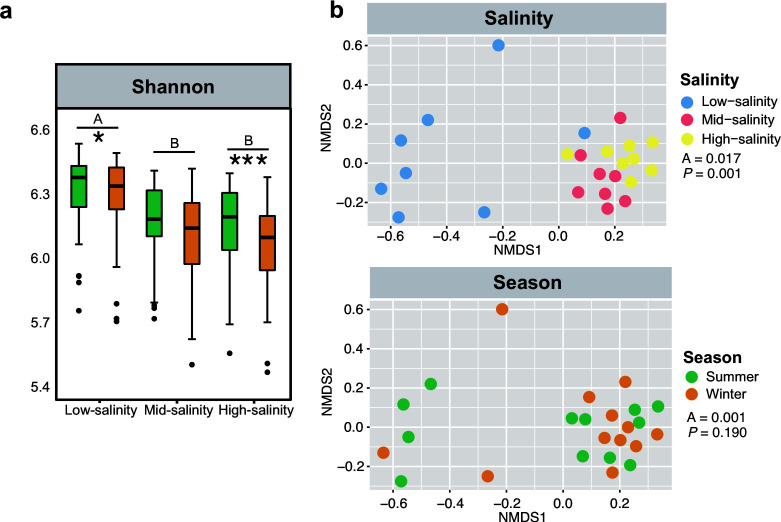
Microbial diversity of Zhenhai Bay sediments along a salinity gradient. (**a**) Boxplots showing Shannon diversity index of sediment microbial communities across low-, mid-, and high-salinity groups in two seasons. Kruskal-Wallis tests were used for multiple-group comparisons and Wilcoxon tests for pairwise seasonal comparisons. Different letters indicate significant differences among salinity groups (*P* < 0.05). Asterisks denote significance levels: *P < 0.05, **P < 0.01, and ***P < 0.001. (**b**) Non-metric multidimensional scaling (NMDS) ordination based on Bray-Curtis distances illustrating beta-diversity patterns. Community dissimilarities were evaluated using ANOSIM; the A statistic quantifies the degree of separation between groups. Detailed statistics for relative abundance and diversity indices are provided in Tables S3 and S4.

Alpha-diversity indices (Shannon, Chao1, and Observed operational taxonomic units [OTUs]) demonstrated that Zhenhai Bay sediments supported taxonomically rich communities (average = 644 OTUs; Shannon index = 6.18), but diversity declined significantly with increasing salinity (Kruskal-Wallis *P* < 0.05; Fig. 1a; Fig. S4a and b; Table S4). Seasonal effects were minor compared with the salinity-driven variation. Rarefaction analysis and high singleton proportions reflect an extensive rare biosphere and the persistence of relic DNA in these sediments, a common feature that explains the high Chao1 estimates (Fig. S4c). Nevertheless, the core community was well represented, with Good’s coverage reaching 80.2% for the non-singleton taxa. Non-metric multidimensional scaling based on Bray-Curtis distances revealed clear clustering by salinity regime (analysis of similarities [ANOSIM], A = 0.017, *P* = 0.001), whereas summer-winter differences were not significant (A = 0.001, *P* = 0.190; Fig. 1b). Taxa within *Crenarchaeota*, *Gammaproteobacteria*, and *Bacteroidia* exhibited positive associations with salinity, while *Gemmatimonadota* and *Verrucomicrobiota* were more abundant under lower-salinity conditions (Fig. S5). Overall, these results demonstrate that salinity acts as a major environmental-filtering force, shaping the diversity and composition of sediment microbial communities in coastal sediments.

### Salinity-driven diversification of aerobic hydrocarbon-degrading genes in coastal sediments

The non-redundant gene catalog of Zhenhai Bay sediments comprised 12,980,628 protein-coding sequences, of which 81.1% were successfully annotated against the eggNOG database (Fig. S6a through c). Among these, bacterial genes dominated (83.7%), while archaeal genes represented only ~1% of the catalog (Fig. S6d), confirming that bacteria constitute the principal drivers of metabolic potential in this estuarine system. From this catalog, we identified a dereplicated set of 509 aerobic hydrocarbon-degrading genes, spanning 10 distinct functional types involved in alkane and aromatic hydrocarbon oxidation (Fig. S7 to S16; Table S5). These included alkane monooxygenases (AlkB, *n* = 184; CYP153, *n* = 281; LadA_alpha, *n* = 19) and ring-hydroxylating dioxygenases (TmoA/BmoA, *n* = 1; TmoE, *n* = 5; DmpO, *n* = 1; MAH_beta, *n* = 9; NdoB, *n* = 5; NdoC, *n* = 1; and non-NdoB type, *n* = 3). AlkB and CYP153 encode key enzymes in the aerobic oxidation of short-chain alkanes, while LadA catalyzes the activation of long-chain alkanes (up to C_36_) (23–26). Ring-hydroxylating dioxygenases such as Ndo (naphthalene dioxygenase), MAH (monoaromatic dioxygenase), Tmo, and DmpO mediate the NADH-dependent hydroxylation of mono- and polyaromatic hydrocarbons (27, 28).

**Fig 2:**
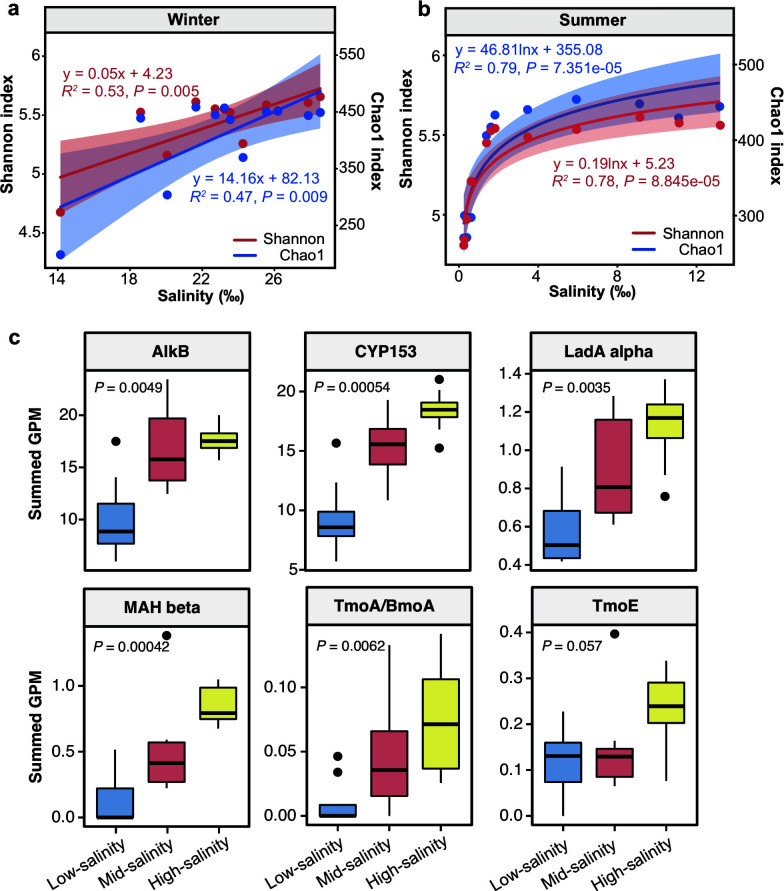
Distribution patterns of aerobic hydrocarbon-degrading genes in Zhenhai Bay sediments. (**a and b**) Relationships between functional diversity of hydrocarbon-degrading genes and salinity in winter (**a**) and summer (**b**). Fitted regression curves for diversity (Shannon index, red) and richness (Chao1 index, blue) are shown with 95% confidence intervals. (**c**) Boxplots showing summed gene abundances (GPM, genes per million) of aerobic hydrocarbon-degrading gene types across three salinity groups. Significance of group differences was determined by Kruskal-Wallis tests (*P < 0.05, **P < 0.01, and ***P < 0.001). Detailed annotations and abundance values are provided in Table S5.

The diversity of these hydrocarbon-degrading genes showed a significant positive association with salinity (Fig. 2a and b; Table S6). Regression analyses revealed significant relationships between salinity and both functional diversity (Shannon index) and gene richness (Chao1 index) across seasons (Shannon index: *R*^2^ = 0.53, *P* = 0.005 in winter, *R*^2^ = 0.78, *P* < 10^−4^ in summer; Chao1 index: *R*^2^ = 0.47, *P* = 0.009 in winter, *R*^2^ = 0.79, *P* < 10^−4^ in summer). Gene abundance comparisons across salinity groups further supported these trends (Fig. 2c). Alkane monooxygenase genes (*alkB*, *cyp153*, and *ladA_alpha*) and several ring-hydroxylating dioxygenase genes (*tmoA/bmoA*, *MAH_beta*, and *tmoE*) were significantly more abundant in high-salinity sediments (Kruskal-Wallis *P* < 0.05), while no significant seasonal differences were observed for hydrocarbon degradation genes (Fig. S17 and 18). Among these, *alkB* exhibited the highest normalized abundance (mean = 1.6 genes per million [GPM]), underscoring its ecological importance in coastal carbon turnover. The enrichment of these genes under saline conditions indicates a functional selection, where the microbial community may harbor a higher metabolic potential for hydrocarbon oxidation in high-salinity habitats.

### Environmental and spatial determinants of hydrocarbon-degrading gene biogeography

**Fig 3:**
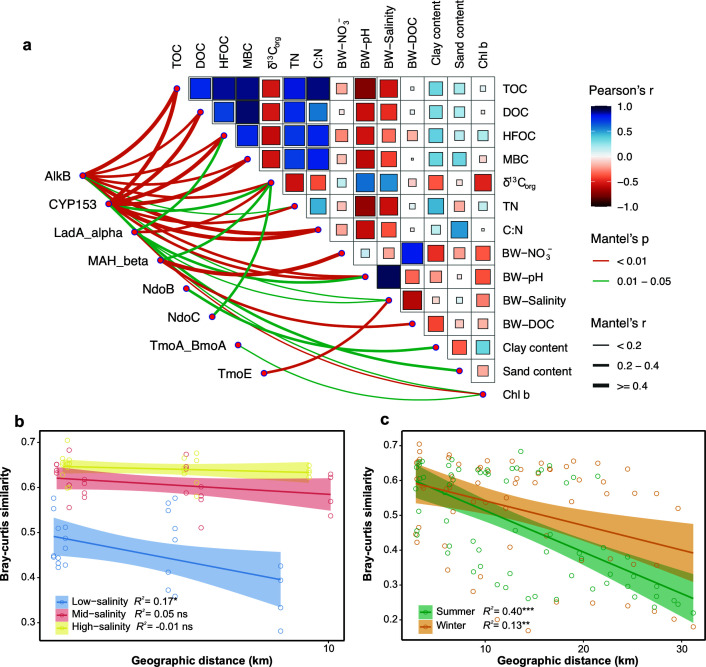
Environmental and spatial drivers of aerobic hydrocarbon-degrading genes in Zhenhai Bay sediments. (**a**) Pairwise correlations among major environmental variables (heatmap) and their Mantel-test associations with hydrocarbon-degrading genes. Color scale indicates Pearson’s *r* (red = negative, blue = positive). Line width represents Mantel’s *r*; color denotes significance (red <0.01 and green <0.05). (**b and c**) Distance-decay relationships (DDRs) between Bray-Curtis similarity and geographic distance across salinity zones (**b**) and seasons (**c**). Shaded areas denote 95% confidence intervals; *R*^2^ values indicate regression fit. Detailed correlation data are provided in Table S7.

To disentangle the key factors shaping the distribution of aerobic hydrocarbon-degrading genes, we assessed the relationships between gene abundances and environmental parameters of both sediments and bottom water (Fig. 3a; Fig. S19; Tables S2 and S7). Mantel tests revealed salinity and pH as the strongest correlates of gene distribution, highlighting their central roles in structuring the hydrocarbon-degrading potential along the estuarine gradient.

Among organic-carbon variables, significant associations were detected with δ^13^Corg, dissolved organic carbon (DOC), heavy fraction organic carbon (HFOC), microbial biomass carbon (MBC), and total organic carbon (TOC), indicating that microbial communities modulate hydrocarbon-degrading gene abundance in response to both labile and recalcitrant carbon pools (29, 30). These correlations suggest that coastal microbiomes maintain versatile oxidation capacities to exploit diverse carbon substrates under fluctuating salinity regimes. The dominant alkane-oxidizing genes *alkB* and *cyp153* showed pronounced positive correlations with nitrate (NO_3_⁻) concentrations (*P* < 0.05). Pigment-related parameters, chlorophyll b, also exhibited positive associations with hydrocarbon-degrading genes, consistent with a photo-linked microbial coupling observed globally (7). Collectively, alkane-degrading genes exhibited stronger environmental coupling than aromatic-degrading counterparts, implying greater environmental sensitivity of alkane oxidation pathways in estuarine sediments.

To evaluate the role of spatial processes, we examined distance-decay relationships (DDRs) between community similarity (Bray-Curtis) and geographic distance (Fig. 3b and c). A pronounced decline in similarity with distance was observed in the low-salinity zone (*R*^2^ = 0.17*) but not in mid- or high-salinity regions (*R*^2^ < 0.05, ns), indicating higher turnover and environmental heterogeneity in freshwater-influenced sediments. When the entire bay was considered, significant DDRs were evident in both seasons (Summer: *R*^2^ = 0.40***; Winter: *R*^2^ = 0.13**), suggesting that spatial isolation and seasonal variation jointly contribute to the biogeographic structuring of hydrocarbon-degrading functional assemblages. Overall, these findings reveal that spatial distance constrains gene dispersal and turnover, ultimately shaping the metabolic biogeography of coastal microbiomes.

### Hydrocarbon degraders act as potential keystone taxa stabilizing microbial networks along the salinity gradient

A total of 278 species-level metagenome-assembled genomes (MAGs) were reconstructed from the 24 sediment metagenomes, representing 26 bacterial and 2 archaeal phyla (Table S8). Quality assessment showed high completeness (75.5% on average) and low contamination (4.7% on average) across MAGs (Fig. S20a). Taxonomic profiling revealed that most MAGs were affiliated with *Pseudomonadota* (*n* = 82), *Acidobacteriota* (*n* = 33), *Gemmatimonadota* (*n* = 30), and *Chloroflexota* (*n* = 24), which together accounted for over 60% of all genomes (Fig. S20b). Among these, 30 MAGs (10.8%) possessed at least one aerobic hydrocarbon-degrading gene, comprising 87 unique monooxygenases and dioxygenases (Fig. S21a ; Table S9). The majority of these belonged to the alkane degradation pathway, including *alkB* (*n* = 14), *cyp153* (*n* = 61), and *ladA* (*n* = 8), whereas fewer genes encoded aromatic hydrocarbon degradation enzymes (MAH_beta = 2, *tmoA/bmoA* = 1, and *tmoE* = 1). Phylogenetic analyses revealed that CYP153 sequences exhibited extensive taxonomic and sequence diversity, forming eight clusters spanning four phyla (Fig. S21b and c). The 30 hydrocarbon-degrading MAGs were distributed among *Pseudomonadota* (*n* = 23), *Myxococcota_A* (*n* = 4), *Actinomycetota* (*n* = 2), and *Desulfobacterota_B* (*n* = 1), with *Gammaproteobacteria* (*n* = 20) dominating and harboring the full complement of alkane and aromatic degradation genes. Several lineages, including HTCC2089 and Ga0077536, encoded enzymes for both substrate types (7), and nearly half of the degraders carried multiple copies of degradation genes, indicating a potential gene-dosage strategy to maintain metabolic resilience under saline stress.

**Fig 4:**
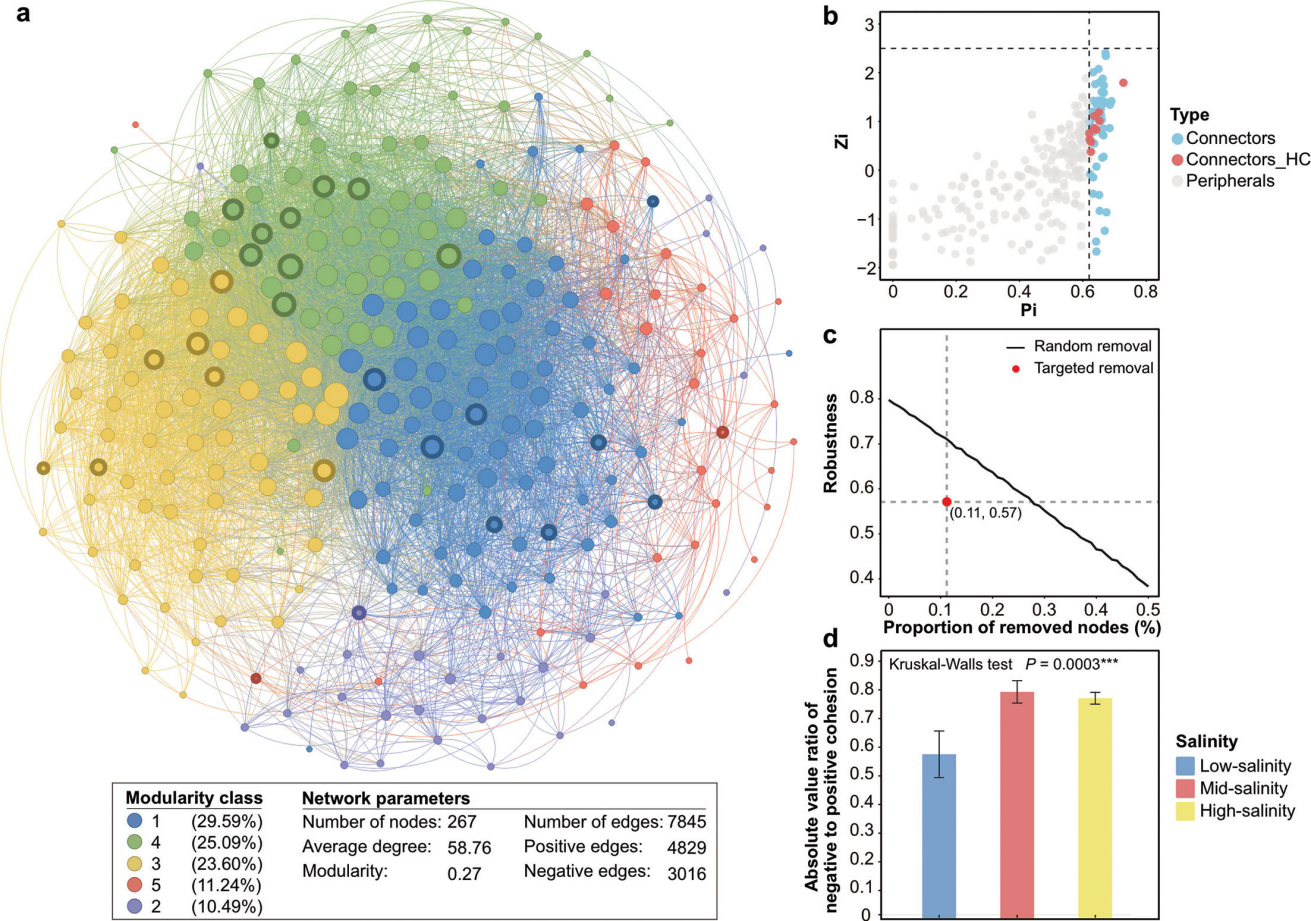
Network centrality of hydrocarbon-degrading bacteria in the Zhenhai Bay microbial community. (**a**) Co-occurrence network of the Zhenhai Bay microbial community. Modules are shown in distinct colors; hydrocarbon-degrading MAGs are highlighted with bold circles. (**b**) Identification of keystone nodes based on within-module (Zi) and among-module (Pi) connectivity. (**c**) Network robustness after random versus targeted removal of hydrocarbon degraders. (**d**) Network stability across salinity gradients measured by the absolute ratio of negative-to-positive cohesion (Kruskal-Wallis *P* = 0.0003). Detailed information on MAGs, genes, and network statistics is provided in Tables S9 through S11.

Co-occurrence network analysis uncovered that hydrocarbon-degrading bacteria occupy central positions in the microbial interaction web of Zhenhai Bay (Fig. 4a through d; Tables S10 and S11). Among the 30 degraders, 10 taxa across 4 phyla were classified as connector hubs (keystone nodes) based on within-module (Zi) and among-module (Pi) connectivity (Fig. 4b) (31, 32). Targeted node-removal experiments demonstrated that deleting these degraders dramatically reduced network robustness (robustness = 0.57) compared with random node loss (0.71; Fig. 4c), confirming that hydrocarbon degraders are pivotal to network stability and information flow in saline sediments.

Salinity exerted a strong influence on network topology. The high-salinity community exhibited a markedly denser and more modular structure (226 nodes and 1,664 edges) compared with the low-salinity community (217 nodes and 715 edges; Fig. S22), despite lower alpha diversity (Fig. 1a). This suggests that high-salinity ecosystems may maintain stability not through taxonomic richness, but potentially via increased ecological connectivity and potential functional coupling among fewer but more strongly associated taxa. Cohesion analysis supported this interpretation: the absolute ratio of negative-to-positive cohesion increased significantly with salinity (0.77 vs 0.57; Kruskal-Wallis *P* = 0.0003; Fig. 4d), suggesting stronger antagonistic-synergistic balance and higher resilience in salt-adapted networks. Seasonally, summer networks displayed greater complexity (13,624 edges) than winter (7,338 edges; Fig. S23), reflecting increased ecological co-variation and more structured community associations during warmer, nutrient-richer periods.

### Halotolerance and energy metabolism in hydrocarbon-degrading microbes

**Fig 5:**
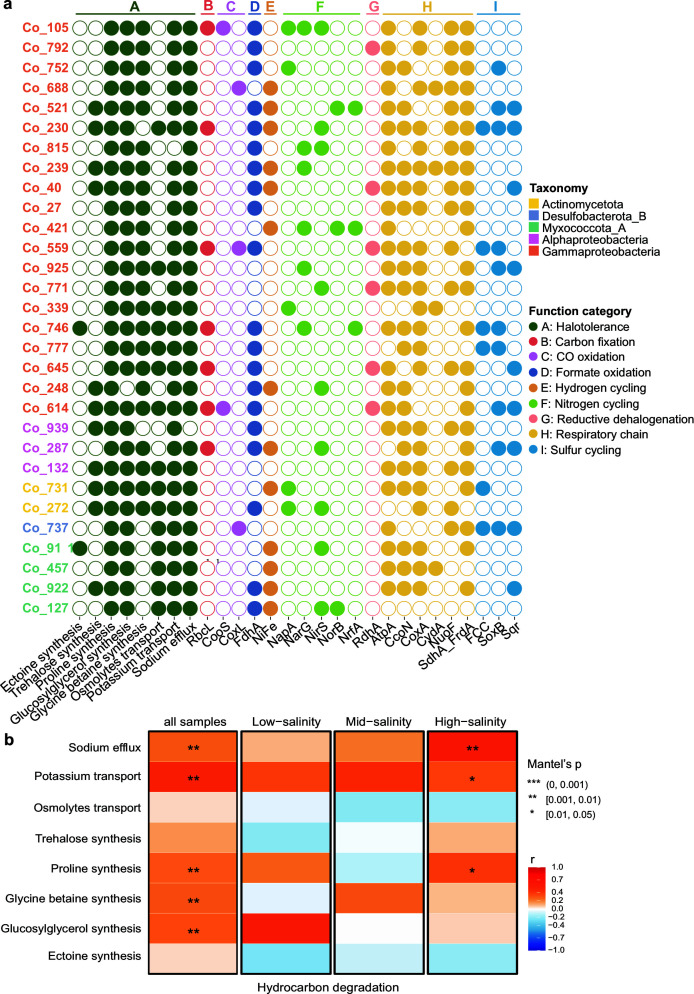
Diverse metabolic functions and halotolerance traits detected in hydrocarbon-degrading bacteria. (**a**) Metabolic functions of 30 hydrocarbon-degrading MAGs spanning four phyla. Colored circles indicate the presence of genes across nine functional categories (A-I): A, halotolerance; B, carbon fixation; C, carbon-monoxide oxidation; D, formate oxidation; E, hydrogen cycling; F, nitrogen cycling; G, reductive dehalogenation; H, respiratory chain; and I, sulfur cycling. Taxonomic affiliations are color-coded by phylum. (**b**) Correlations between hydrocarbon-degrading genes and halotolerant genes across all samples and salinity groups. Color scale represents Pearson’s *r*, with red and blue denoting positive and negative correlations, respectively. Asterisks indicate significance: ***P < 0.001, **P < 0.01, and *P < 0.05. A complete list of functional genes is provided in Tables S12 and 13.

Across halophilic and halotolerant microorganisms, two principal osmoadaptation strategies are commonly employed to maintain intracellular osmotic equilibrium: ion accumulation and biosynthesis or uptake of compatible solutes (33–35). To investigate how hydrocarbon degraders cope with salt stress, we annotated osmoadaptation-related marker genes across the 30 aerobic hydrocarbon-degrading MAGs. These microbes displayed diverse halotolerance repertoires, including genes for the synthesis or transport of ectoine, trehalose, proline, glucosylglycerol, and glycine betaine, together with Na^+^/K^+^ ion transport systems (36, 37) (Fig. 5a; Table S12). Among the organic osmolyte biosynthesis mechanisms, genes for proline, glucosylglycerol, and glycine betaine biosynthesis were the most numerous in the genomes, consistent with previous reports of betaine accumulation in hydrocarbon-degrading *Rhodococcus* strains (38). Nearly all MAGs encoded sodium or potassium efflux pumps, whereas several *Gammaproteobacteria* lineages (e.g., *Woeseiales*, *Xanthomonadales*, and Ga0077536) lacked complete osmolyte transport systems. While this absence could partially be attributed to the inherent incompleteness of MAGs, it may point toward a potential preference for ionic adjustment in these specific groups under saline conditions. Correlation analyses based on the abundance of hydrocarbon-degrading genes and halotolerance genes within the 30 aerobic hydrocarbon-degrading MAGs revealed a strong positive association, particularly under high-salinity conditions (Fig. 5b), with similarly positive correlations observed in both summer and winter samples, although the relationship was stronger in summer (Fig. S24). This coupling implies that hydrocarbon oxidation may energize osmoadaptive processes, allowing cells to redirect catabolic energy toward maintaining ionic balance and turgor pressure during salt stress.

As osmoadaptation can impose energetic costs (39), we next examined how hydrocarbon degraders sustain the required energy flux. Functional profiling identified multiple pathways for aerobic respiration and alternative energy generation (Fig. 5a; Table S13). Among the 30 aerobic hydrocarbon-degrading MAGs, nearly all encoded canonical aerobic respiratory complexes (*ccoN*, *coxA*, and *cydA*), and several MAGs carried enzymes for trace-gas oxidation, including the large subunit of group 1 [NiFe]-hydrogenases (33%), carbon-monoxide dehydrogenases (*coxL*, 10%), and RuBisCO (*rbcL*, 23%), conferring the capacity to exploit atmospheric H_2_ and CO and/or to fix carbon. Genes for sulfide and thiosulfate oxidation (*sqr*, *fcc*, and *soxB*) were broadly distributed across taxa, and several MAGs encoded anaerobic energy-yielding routes, such as hydrogenogenic fermentation, nitrogen-oxide reduction, and arsenate respiration (*arsC*). Collectively, these findings demonstrate that hydrocarbon-degrading microbes employ metabolically diverse and energy-efficient strategies, coupling hydrocarbon oxidation with alternative electron-transfer and osmoadaptive systems.

### Horizontal gene transfer and substrate diversification enhance hydrocarbon degradation capacity

Comparative genomic and phylogenetic analyses revealed that most hydrocarbon degraders in Zhenhai Bay harbor multiple copies of key alkane-oxidizing genes, particularly *alkB* and *cyp153*. To elucidate their evolutionary dynamics, we performed gene-species tree reconciliation to infer duplication, loss, and HGT events of aerobic alkane monooxygenases. The resulting reconciled topology highlighted frequent horizontal transfer and duplication of *alkB* and *cyp153* genes across phylogenetically distant bacteria (Fig. S25; Table S14). The most extensive gene transfer and duplication events were associated with four genomes within the HTCC2089 lineage of *Gammaproteobacteria*, with closely related *cyp153* homologs also detected in *Myxococcota_A*, *Desulfobacterota_B*, and *Actinomycetota*. Within *Sphingomonadales*, *cyp153* copies related to the HTCC2089 lineage underwent repeated duplication, indicating that horizontally acquired genes are retained and expanded following acquisition. Similarly, a *Halieaceae* genome encoded two *alkB* and five *cyp153* genes, several of which were phylogenetically related to homologs in other hydrocarbon degraders.

**Fig 6:**
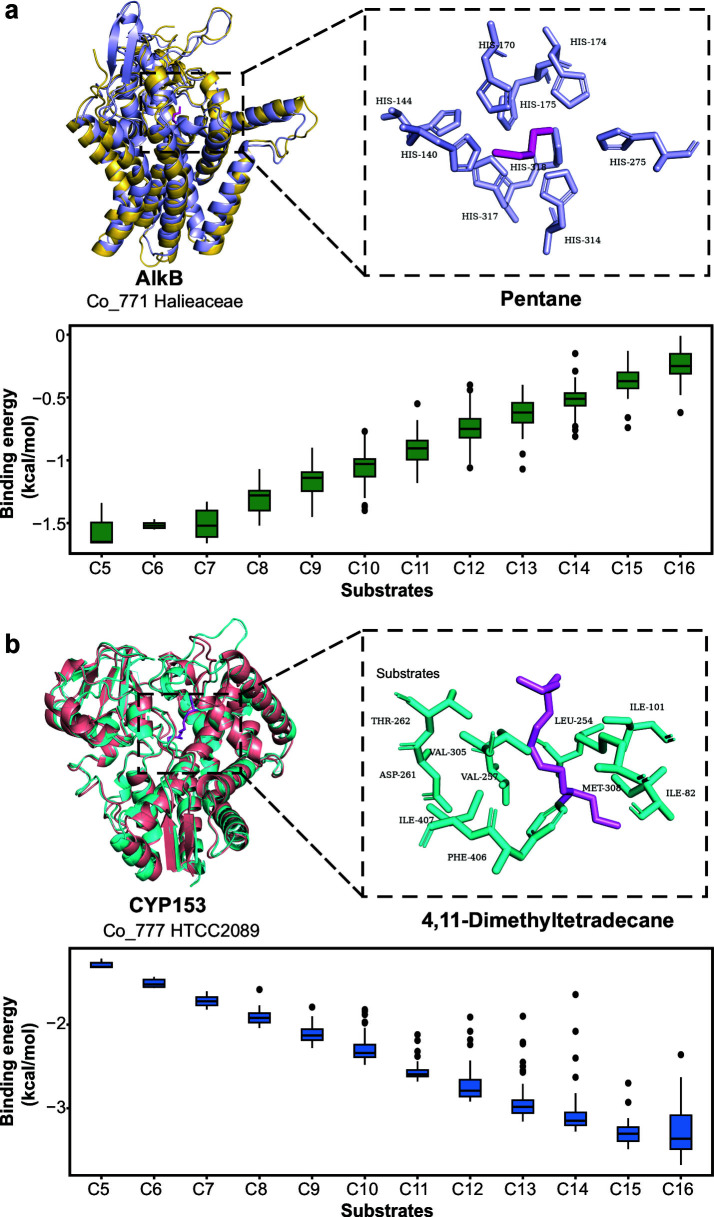
Substrate preferences of hydrocarbon-degrading genes AlkB and CYP153. (**a**) Structural comparison between AlphaFold-predicted AlkB from *Halieaceae* (Co_771) and the experimentally verified AlkB (PDB 8SBB, yellow). The inset shows the active-site binding of AlkB with pentane (violet sticks). Boxplots depict binding energies (kcal mol^−1^) of AlkB for C_5_–C_16_ substrates. (**b**) Structural comparison between AlphaFold-predicted CYP153 from HTCC2089 (Co_777, cyan) and the experimentally determined CYP153 (PDB 6HQD, salmon). The inset highlights interaction with 4,11-dimethyltetradecane (violet sticks). Boxplots show binding energies of CYP153 for C_5_–C_16_ substrates. Detailed statistics for gene events and molecular docking are provided in Tables S14 and S15.

Given that multiple gene copies may exhibit distinct substrate specificities or expression patterns (7, 40), we further investigated the functional range of substrate oxidation through molecular docking of 21 AlkB and CYP153 proteins from *Pseudomonadota* against a panel of linear and branched alkanes (C_5_–C_16_) (41) (Fig. 6a and b; Table S15). The enzymes showed broad predicted substrate recognition, with binding energies spanning −0.01 to −4.39 kcal mol^−1^. AlkB enzymes showed a tendency toward short-chain alkanes (C_5_–C_8_), whereas CYP153 exhibited relatively higher predicted affinity for longer-chain substrates (C_14_–C_16_). Specifically, AlkB from *Halieaceae* and HTCC2089 showed strong predicted binding to pentane (−1.66 and −1.15 kcal mol^−1^, respectively; Fig. 6a) (42), while horizontally transferred CYP153 from HTCC2089 was predicted to bind multiple linear and branched alkanes, averaging −2.63 kcal mol^−1^ for C_5_–C_16_ (Fig. 6b), comparable to the experimentally validated *Pseudomonas* sp. 19-rlim CYP153 (−3.11 kcal mol^−1^, PDB 6HQD) (43). Moreover, distinct CYP153 paralogs within the same genome exhibited divergent predicted substrate preferences (Fig. S26), suggesting functional diversification among gene copies. Together, these results reveal that frequent horizontal transfer and gene duplication, coupled with substrate-specific diversification, may expand the metabolic repertoire of hydrocarbon degraders.

## DISCUSSION

Salinity represents one of the most pervasive environmental drivers in coastal ecosystems, fundamentally influencing microbial diversity, metabolism, and ecological function (11, 13, 14). In this study, we provide an integrative metagenomic and genome-resolved perspective on how salinity regulates the diversity, evolution, and ecological roles of aerobic hydrocarbon-degrading microbes in Zhenhai Bay sediments. Our results reveal a multifaceted picture in which salinity governs not only the taxonomic and functional structure of hydrocarbon-degrading communities but also their metabolic potentials, network stability, and evolutionary innovation.

Across the natural salinity gradient, we identified 10 types of aerobic hydrocarbon-degrading genes, encompassing both alkane monooxygenases (*alkB*, *cyp153*, and *ladA*) and ring-hydroxylating dioxygenases (*ndo*, *tmo*, and *dmpO*). The alkane monooxygenases (484 of 509 sequences) were numerically dominant, indicating that aerobic alkane degradation represents the primary hydrocarbon turnover route in Zhenhai Bay sediments, consistent with observations from global oceanic metagenomes (7). Their relative abundance and functional diversity increased significantly toward high-salinity regions, suggesting that salinity is a key factor shaping the functional structure of the community, where the enrichment of hydrocarbon-degrading genes likely reflects a taxonomic turnover toward specific halophilic lineages with diverse metabolic potentials. This enrichment pattern is consistent with global oceanic surveys showing preferential expansion of alkane-degrading genes in saline and hydrocarbon-enriched habitats (7).

Mantel and distance-decay analyses revealed that hydrocarbon-degrading gene composition is shaped by both local physicochemical gradients and regional spatial turnover. Salinity, together with organic carbon parameters (δ¹³C_org, DOC, TOC, and HFOC), emerged as a major determinant of gene composition, highlighting a coupled response of degraders to osmotic pressure and carbon-energy availability (44, 45). In addition, the observed associations with nitrate and chlorophyll b suggest that nitrogen availability and photo-associated microbial processes may further modulate hydrocarbon degradation potential in coastal sediments. Consistent with previous studies, nitrate availability has been shown to enhance aerobic hydrocarbon biodegradation by stimulating nitrogen-dependent co-metabolism and microbial biomass growth (46, 47). Stronger distance-decay relationships in low-salinity regions indicate that environmental heterogeneity enhances community differentiation, whereas high-salinity regions harbor more compositionally stable assemblages. These findings align with metacommunity theory, suggesting that environmental filtering dominates in saline habitats, while stochastic processes exert greater influence in less saline zones (48–50). Given that oxygen availability and redox conditions are critical factors for aerobic hydrocarbon degradation, we performed a Partial Mantel test controlling for multiple redox- and nutrient-related variables. Salinity remained significantly correlated with hydrocarbon-degrading gene abundance (*r* = 0.1507, *P* = 0.05), suggesting that the observed patterns are associated with salinity beyond the effects of oxygen or redox variations. However, it should be noted that this study focused on surface sediments across spatially distinct sites rather than sediment depth profiles; thus, the influence of vertical oxygen or redox stratification remains to be further explored.

Co-occurrence network analysis further demonstrated that hydrocarbon degraders, particularly *Gammaproteobacteria*, act as potential keystone taxa maintaining network stability and functional integrity. Targeted removal of these taxa markedly decreased network robustness, confirming their structural importance. Although high-salinity sediments exhibited lower alpha diversity, their microbial networks were denser and more cohesive, suggesting that increased interaction complexity compensates for reduced taxonomic richness (51). Such cooperative and competitive dynamics likely enhance the resilience of hydrocarbon-degrading consortia under osmotic stress. Nevertheless, as these network-based inferences are primarily statistical, future studies incorporating transcriptomics or stable isotope probing are needed to further validate the ecological significance and metabolic activities of these potential keystone taxa within the community.

Osmoregulation in saline environments can impose substantial energetic costs, depending on the osmoadaptive strategy employed, such as compatible solute synthesis or active ion transport to maintain osmotic balance. The hydrocarbon-degrading bacteria we identified encoded multiple halotolerance mechanisms, including biosynthesis and uptake of compatible solutes (ectoine, proline, glycine betaine, and glucosylglycerol) and Na^+^/K^+^ ion transport systems. Positive correlations between halotolerance and hydrocarbon-degrading genes imply that catabolic energy from hydrocarbon oxidation may fuel osmoadaptive processes, representing a potential link between carbon metabolism and salt tolerance, as supported by studies showing hydrocarbon-degrading microbes using energy from hydrocarbons to cope with high salinity (52, 53). Furthermore, the widespread presence of genes for aerobic respiration, hydrogen and CO oxidation, and sulfur and nitrogen transformations suggests that degraders likely employ diverse bioenergetic strategies to sustain activity under fluctuating redox and salinity regimes.

Gene-species reconciliation analyses revealed pervasive predicted HGT and gene duplication events of *alkB* and *cyp153*, particularly among lineages such as HTCC2089 and *Halieaceae*. These duplication and transfer patterns underscore the metabolic importance of hydrocarbon-degrading genes and their role in niche adaptation (54), while we acknowledge that MAG completeness and potential contamination may influence the inference of HGT patterns. Molecular docking further demonstrated that AlkB was predicted to preferentially oxidize short-chain alkanes, whereas CYP153 shows high predicted affinity for long-chain substrates. Notably, paralogous CYP153 copies within the same genome exhibited distinct predicted substrate-binding profiles, indicating functional divergence following duplication (55). Such evolutionary innovation through gene mobility and diversification enhances the ecological adaptability of hydrocarbon-degrading bacteria under selective pressure from salinity and substrate heterogeneity.

### Conclusions

Taken together, our results demonstrate that salinity not only shapes the diversity and distribution of hydrocarbon-degrading microbes but also drives their adaptation through metabolic potential, gene acquisition, and community reorganization. Hydrocarbon degraders represent functionally important members of the microbial community that are associated with osmoadaptation, carbon turnover, and community stability in coastal sediments. These insights extend the conceptual understanding of how environmental gradients reconfigure microbial metabolism and provide a basis for exploring microbial responses to increasing coastal salinization. From an applied perspective, the identification of metabolically flexible and salt-tolerant degraders provides valuable guidance for the design of bioremediation strategies in saline and estuarine environments, where conventional degradation processes are often limited by osmotic stress. Our study thus bridges microbial evolutionary ecology and environmental biotechnology, revealing how life adapts to, and thrives in salinity-driven constraints.

## MATERIALS AND METHODS

### Study area and sampling

Zhenhai Bay, located in Guangdong Province, China, lies on the coast of the South China Sea to the west of the Pearl River Delta (Fig. S1). This region experiences a humid subtropical climate with more than 1,700 h of annual sunshine, an average annual precipitation of 2,183.3 mm, mean temperatures ranging from 21.3°C to 22.8°C, and a perennial average relative humidity of 86% (56, 57). This semi-enclosed, shallow bay has depths between 0 and 10 m, encompassing an area of about 156 km² and stretching roughly 27 km in length. It receives substantial inputs of freshwater, sediment, and solutes from a large watershed area of approximately 2,832 km². This results in a marked salinity gradient (0.17–28.54 PSU) and a dominance of fine mud sediments in the ecosystem (57). Rapid population growth and urbanization have led to numerous eco-environmental problems in the Zhenhai Bay, including widespread eutrophication and increased occurrences of harmful algal blooms (58, 59).

The sampling stations span from 21.785 to 22.053°N in latitude and 112.371 to 112.487 °E in longitude (Fig. S1; Table S1). Sediments consisting of fine mud were collected from 12 sites of the Zhenhai Bay using a core cylinder, a PVC pipe handle, and a one-way valve in winter (26 January 2019) and summer (4 August 2019). Based on *in situ* salinity measurements, the 12 sites were grouped into three salinity categories: low-, mid-, and high-salinity (Fig. S1 and S2; Table S2). At each station, triplicate surface (0–5 cm) sediments were taken from the cores and sealed immediately with airtight, acid-cleaned plastic bags. Bottom water samples were collected in polyethylene bottles and filtered through 0.2 μm filters (Millipore, Bedford, USA). All of the samples were transported on ice to the laboratory. In the laboratory, bottom water samples were stored at −20°C. Sediments in each station were immediately mixed thoroughly under a helium (He) condition, and subsequently divided into three individual parts, and stored at 4°C, −20°C, and −80°C for MBC measurement, other physicochemical properties determination, and DNA extraction, respectively.

### Determination of environmental parameters

Temperature, salinity, dissolved oxygen, pH, and water depth were determined *in situ* with a YSI Professional Plus Multi-parameter Meter (Yellow Springs, OH, USA) at all sites (Fig. S2). Sediment water contents were calculated gravimetrically from fresh sediment dried at 60°C to a constant value (60). Water nutrient samples were used to measure ammonium (NH_4_^+^), nitrite (NO_2_^−^), and nitrate (NO_3_^−^) by a continuous flow nutrient analyzer (Futura, Alliance, France). Sediment exchangeable NH_4_^+^, NO_2_^–^, and NO_3_^–^ were extracted using 2 M KCl, and measured the same way as the water nutrient samples, except for using 2 M KCl instead of Milli-Q water when preparing the standard curve solution. Ferric oxides were extracted from 1 g of fresh sediment by a mixture of both 0.5 M hydrochloric acid (HCl) and 0.25 M hydroxylamine hydrochloride (bubbled with N_2_ prior to the extraction) and analyzed with the ferrozine-based colorimetric method (61). Sediment grain size samples were pretreated by 20% hydrogen peroxide (H_2_O_2_) and 15% HCl solutions in turn to oxidize organic matter and remove carbonate completely, respectively, and then measured by LS 13320 Laser grain sizer (62). TOC and total nitrogen (TN) samples were treated with 1 M HCl to remove carbonate completely, and determined by an Elemental analyzer (Vario EL Cube, Germany). After the removal of inorganic carbon with 1 M HCl, stable isotope ratios (δ^13^C_org_ and δ^15^N) of sediments were analyzed with a stable isotope ratio mass spectrometer (MAT 253, Thermo Fisher Scientific, Dreieich, Germany). Sediment DOC contents were extracted with Milli-Q water and determined with a TOC-TN analyzer (Shimadzu, Kyoto, Japan). Sediment easily oxidized carbon contents were determined using a spectrophotometer colorimetry after oxidation with 333 mM KMnO_4_ (63). HFOC and light fraction organic carbon contents were determined using the density method (64). MBC was measured by the chloroform fumigation-extraction method (65). Sediment pigments were extracted by 80% (vol/vol) acetone at 4°C for 24 h in darkness. Extracts were centrifuged at 2,200 *g* for 10 min, and absorbance was measured using a Shimadzu UV-2550 spectrophotometer (Kyoto, Japan) at 664, 649, and 470 nm. Sediment chlorophyll a (Chl a), chlorophyll b (Chl b), and carotenoid contents were calculated using previously published equations (66).

### Metagenome sequencing and analysis

Genomic DNA extraction of 24 sediment samples was performed using PowerSoil DNA Isolation Kits (MO BIO Laboratories). Then, metagenomic sequencing was performed on the Illumina Novaseq6000 high-throughput sequencing platform and obtained a total of 150.6 GB of raw sequencing data. Using the Read_QC module of metaWRAP (v1.3.2) (67), the raw reads were quality controlled to obtain 149.9 GB of clean reads. Then, individual assembly and co-assembly of the clean reads for each sediment sample were performed using Megahit (v1.1.3, default parameters) (68). All assemblies (*n* = 25) were binned using the binning module (–metabat2 –maxbin2 –concoct), and the three bin sets for each assembly were consolidated into a final bin set using the Bin_refinement module (parameters: –c 50 –x 10) within metaWRAP (67).

Contigs longer than 1,000 bp from all 25 assemblies were used to generate 12,980,628 protein clusters using Prodigal (v2.6.3) (69). These sequences were then clustered at 95% amino acid identity using CD-HIT (v4.8.1) with parameters set to -c 0.95 -aS 0.9 -g 1 -d 0 (70). The non-redundant amino acid sequences were functionally annotated via eggNOG-mapper (v2.1.9) with default settings and referencing the eggNOG database (v5.0.2) (71, 72). Taxonomic assignments for each non-redundant sequence were performed using MMseqs2 taxonomy (v13.45111; parameter: easy-taxonomy --tax-lineage 1) with the GTDB R214 as the reference database (73, 74).

Based on 95% average nucleotide identity, 382 MAGs obtained from the 25 assemblies were dereplicated using the dRep software (v2.5.4) (75), resulting in 278 representative MAGs with ≥50% estimated completeness and ≤10% contamination (MIMAG medium-quality standards). A total of 107,351 genes were predicted from 278 non-redundant MAGs using the Prodigal software. The quality of these MAGs was assessed using CheckM (v1.0.7) with default parameters (76). Taxonomic lineage for each non-redundant MAG was assigned using GTDB-Tk (v2.3.2) based on the GTDB R214 reference database (74, 77). This tool ensures taxonomic accuracy by placing MAGs into a reference tree based on concatenated marker genes (120 for Bacteria; 122 for Archaea) and Relative Evolutionary Divergence, effectively mitigating the influence of minor contamination.

### Functional annotations and phylogenetic analysis

To identify aerobic hydrocarbon-degrading genes, CANT-HYD HMMs (23) were used to annotate 278 MAGs and non-redundant gene catalog with noise cutoff using HMMER (v3.3.2) (78). The hydrocarbon-degrading amino acid sequences from non-redundant MAGs were aligned with reference sequences using MUSCLE (v3.8.1551) (79), and then trimmed with TrimAl software (v1.4.15) (80). The phylogenetic trees were constructed using IQ-TREE (v2.2.3, settings: -m MFP -B 1000) (81) and visualized by iTOL software (v6) (82). For MAGs containing hydrocarbon-degrading genes, we further predicted their metabolic and biogeochemical functions by searching against custom protein databases of representative metabolic marker genes (https://doi.org/10.26180/c.5230745) using DIAMOND. We further used eggNOG-mapper (v2.1.9) (71, 72) to annotate the amino acid sequences of hydrocarbon-degrading MAGs and identify the halotolerant genes based on KO number (50).

### Microbial diversity, community structure, and gene functional diversity

To obtain community profiles for diversity analyses, conserved single-copy ribosomal genes were extracted from the metagenomic data using SingleM software (v0.13.2) (83) and clustered into OTUs. The alpha and beta diversity were then calculated using the vegan package (diversity, estimateR, and metaMDS functions) in R (v4.0.0) based on OTU tables. Phyloflash software was used to extract 16S mitags from the unassembled metagenomes to assess microbial community composition, and the relative abundance of each taxon was calculated at the phylum and class levels (84).

The gene abundances of predicted genes from 278 MAGs and the non-redundant gene catalog across 24 samples were calculated using Salmon (v1.10.2; -validateMappings -meta) and then were normalized to GPM (85). We further calculated the Shannon diversity and richness (Chao1) of the hydrocarbon-degrading genes using the vegan package (diversity and estimateR functions) to reveal their functional diversity.

### Correlation of hydrocarbon-degrading genes with environmental factors

To assess the effect of environmental factors on hydrocarbon degradation, we examined relationships between the abundances of hydrocarbon-degrading genes and the physicochemical properties of sediment and overlying water using Mantel’s test (9,999 permutations) in R software. Distance-decay relationship analyses were implemented to reveal the relationship between the similarity of microbial communities and spatial distance along the Zhenhai Bay. In brief, the “distm” function in Geosphere package was used to calculate spatial distance, and the “vegdist” function in the vegan package can determine the Bray-Curtis similarity of microbial communities.

### Co-occurrence network construction and analysis

Microbial co-occurrence network analyses were performed using the R packages igraph (86) and WGCNA (87). Only MAGs with relative abundances exceeding 0.01% and occurring in more than five samples were included in the analyses. The Spearman correlations |*r|* > 0.7 and False-Discovery Rate corrected *P* < 0.05 were used for co-occurrence network construction. The networks were visualized in Gephi v0.10.1 (88).

We used the Louvain method (89) to detect the modules of the network in Gephi. Each node was then categorized based on its within-module connectivity Zi and among-module connectivity Pi. The node classifications included module hubs (Zi > 2.5 and Pi < 0.62), network hubs (Zi > 2.5 and Pi > 0.62), peripherals (Zi < 2.5 and Pi < 0.62), and connectors (Zi < 2.5 and Pi > 0.62). The calculations of Zi and Pi were performed following the method of Guimerà and Nunes Amaral (90) in R with the package igraph (86). To assess the stability of microbial networks, we measured robustness, defined as the proportion of remaining species after random or targeted removal of hydrocarbon degraders. The network robustness algorithm is based on a method of weighting with the relative abundance of species in this study (32). Random node removal involved randomly eliminating a specified proportion of nodes, ranging from 1% to 50% random node removal. For targeted removal, we calculated the network robustness after removing hydrocarbon degraders. We further calculated the absolute value ratio of negative to positive cohesion to assess the associations among microbial members and the network stability. Cohesion was proposed by Herren and McMahon (91) and was calculated as the total of significant positive or negative correlations between species weighted by species abundance. Network robustness and cohesion were also calculated in R using the matrix of correlation coefficients obtained with the igraph package (86).

### Gene trees and species tree reconciliation

For genomes containing *alkB* and *cyp153* genes, we used GToTree v1.5.22 to identify and align single-copy bacterial ribosomal genes from the 28 genomes we selected (92). Only genomes with more than half of the single-copy genes were retained. The concatenated gene alignments were further used to construct the species tree with IQ-TREE v2.2.3 (81). The gene tree inference was conducted by ParGenes v1.2.0, using the alignments of hydrocarbon-degrading amino acid sequences as inputs (93). Briefly, the phylogeny of *alkB* and *cyp153* genes was constructed using RAxML-NG v1.1.0 with LG+FC+I+G4m model (94). Gene trees and species trees were further reconciled using GeneRax v2.1.3 to identify gene duplication, transfer, loss, and speciation events (95). We used the default settings implemented in GeneRax using a substitution model with LG+G, and a reconciliation scenario distribution was returned with 100 samples to increase accuracy. The output RecPhyloXML files were visualized using ThirdKind v3.6.8 (96).

### Protein modeling and molecular docking

The 3D structures of *alkB* and *cyp153* genes were predicted using AlphaFold Server (https://alphafoldserver.com/) powered by AlphaFold 3.0 (97). The predicted template modeling score was used for measuring the accuracy of the entire structure, and all of them of our structures were above 0.90. For molecular docking, we first obtained the grid center coordinates of pocket boxes based on the active sites using DockingPie v1.2 (98). Then, we used Ledock (v1.0; https://www.lephar.com/) to predict the binding poses of 419 alkanes with different chain lengths (C5-C16) on AlkBs and CYP153s. The chemical structures of 419 alkanes were collected from PubChem database (https://pubchem.ncbi.nlm.nih.gov/). All structures were visualized and exported using PyMOL v2.5.2 (http://www.pymol.org).

### Statistical analyses

Statistical analyses were performed using R (v4.0.0). To compare multiple groups, Kruskal-Wallis rank-sum tests with *χ*^2^ corrections were utilized. The Wilcoxon tests were employed for comparisons between two individual groups. Linear and logarithmic regression analyses were used to fit the data and predict the linear correlation between gene functional diversity and salinity, spatial distance, and the Bray-Curtis similarity of microbial communities. ArcGIS (v10.2), Corel Draw X6, and R (v4.0.0) software were used for graphics.

## Data Availability

All raw metagenomes and metagenome-assembled genomes were deposited to the National Center for Biotechnology Information (NCBI) Sequence Read Archive under BioProject no. PRJNA739379. Protein sequences, protein structures, and phylogenetic trees of hydrocarbon-degrading genes have been uploaded to Figshare (https://doi.org/10.6084/m9.figshare.28551251).

## SUPPLEMENTAL MATERIAL

Supplemental Figures msphere.00369-26-s0001.pdf Figures S1–S26.

Supplemental Tables msphere.00369-26-s0002.xlsx Tables S1–S15.

ASM does not own the copyrights to Supplemental Material that may be linked to, or accessed through, an article. The authors have granted ASM a non-exclusive, world-wide license to publish the Supplemental Material files. Please contact the corresponding author directly for reuse.
