# Supplementary material for: Salinity-driven microbial adaptation of hydrocarbon-degrading communities in coastal sediments
Source: mSphere. 2026 Jul 2;11(7):e00369-26. doi: 10.1128/msphere.00369-26 (PMC13410759; doi:10.1128/msphere.00369-26)
Supplement: Supplemental Figures — Figures S1–S26. [file msphere.00369-26-s0001.pdf]

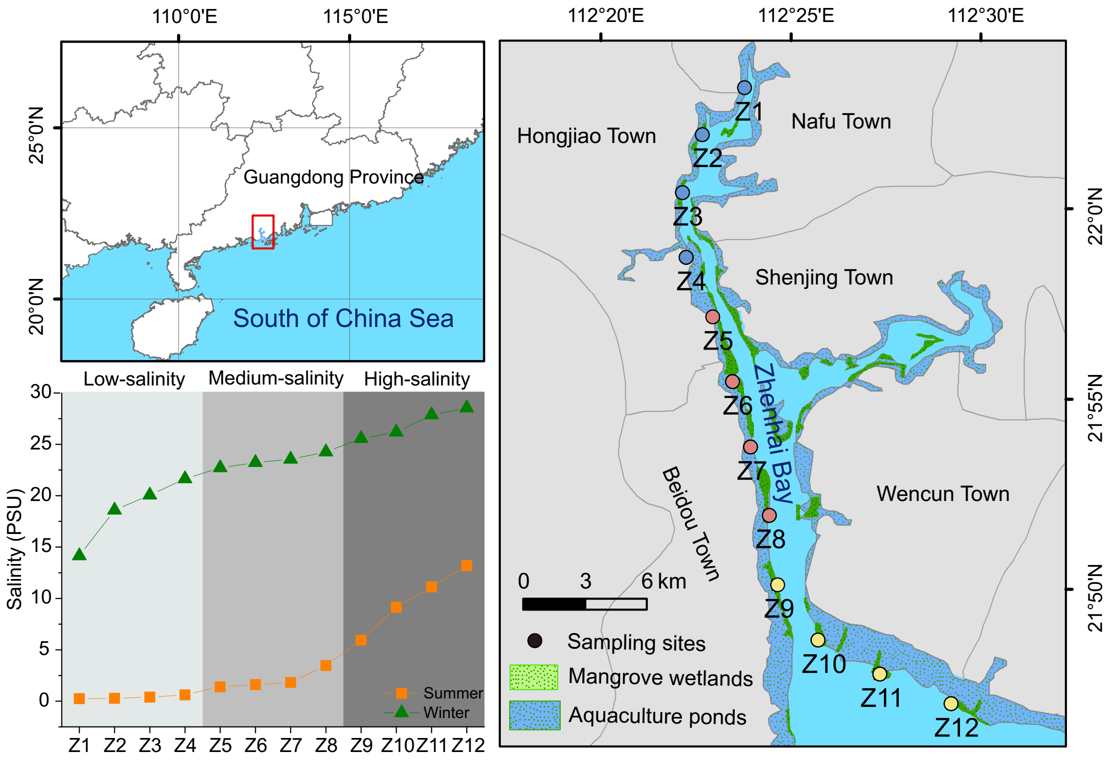

Supplementary Figure 1 Distribution map of sampling sites in Zhenhai Bay. The map was drawn using the ArcGIS v10.2. The salinity groups are highlighted: low-salinity, blue; mid-salinity, red; high-salinity, yellow.

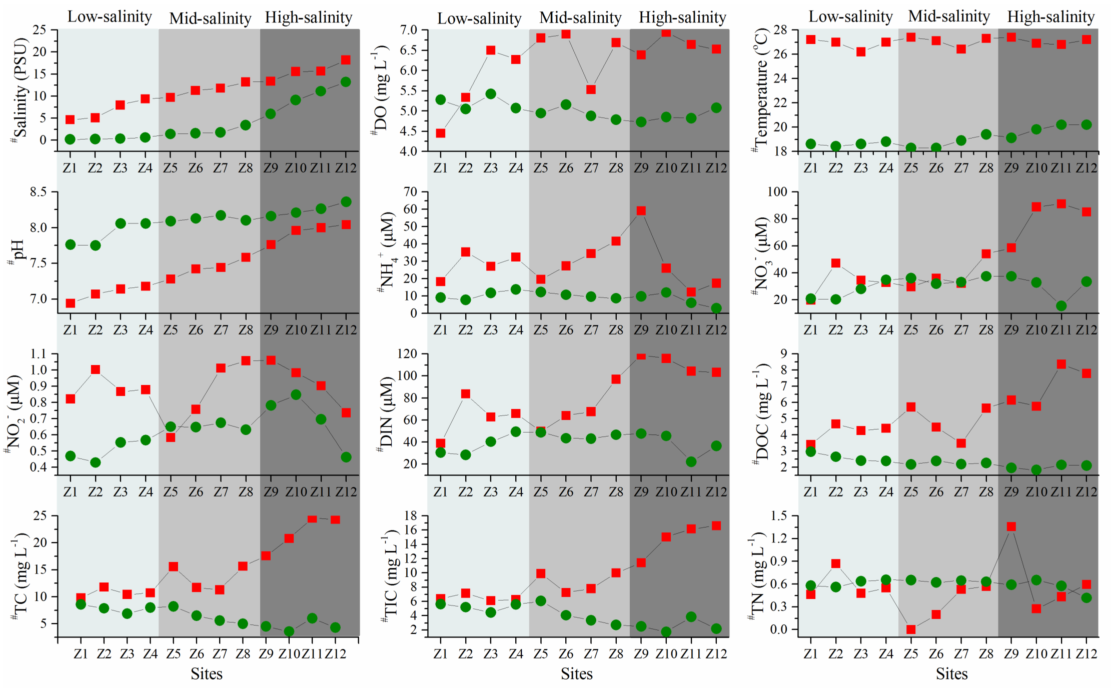

Supplementary Figure 2 Environmental parameters of sampling sites in Zhenhai Bay. The samples collected from the same season are highlighted in the same colors (Winter, red; Summer, green). Detailed statistics for environmental factors of each sample can be found in Supplementary Table 2.

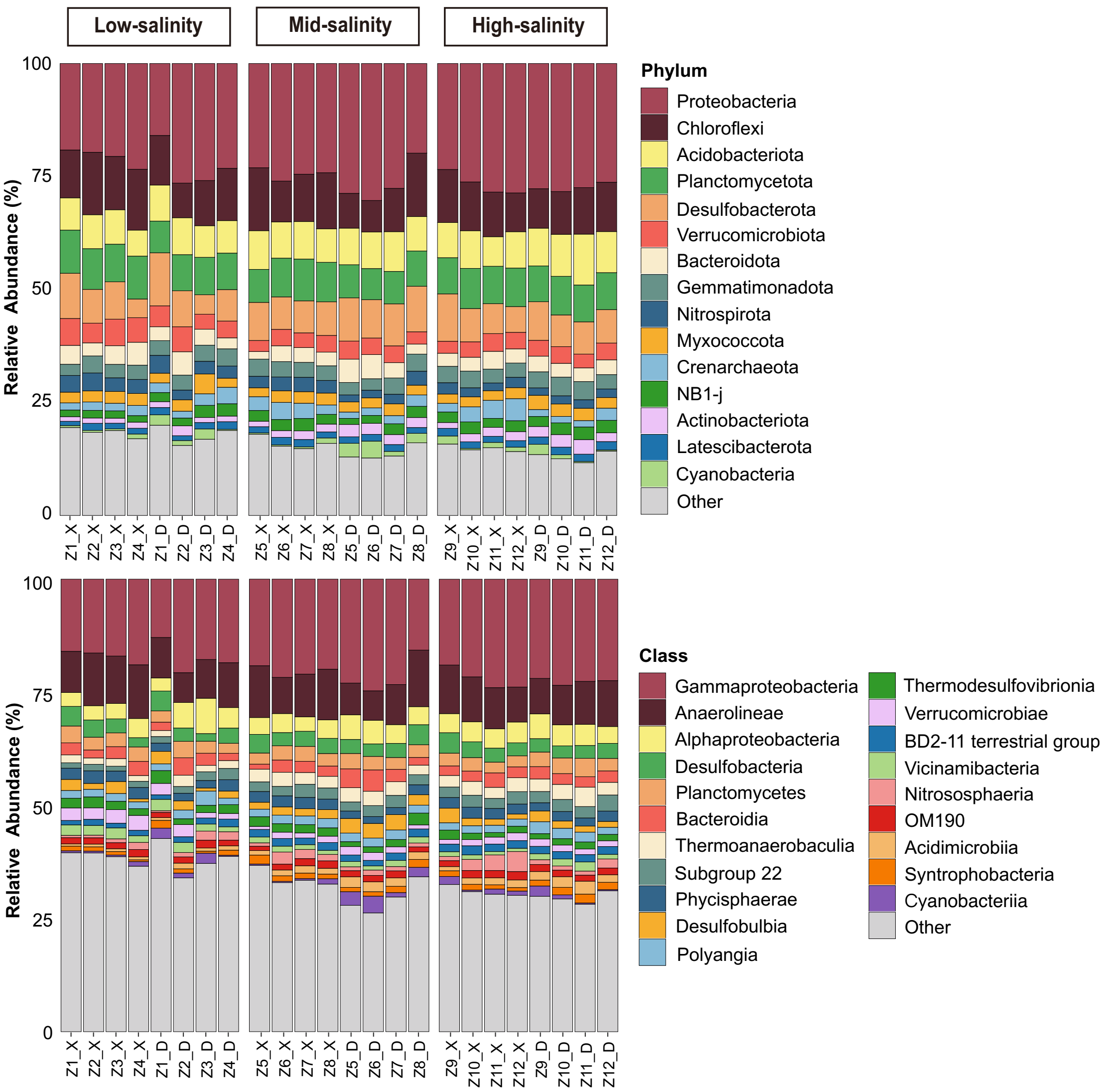

Supplementary Figure 3 Microbial community structure of 24 Zhenhai Bay sediments along a salinity gradient. Relative abundance of the detected bacterial and archaeal OTUs at phylum and class levels. Detailed statistics for the relative abundance of each OTUs in each sample can be found in Supplementary Table 3.

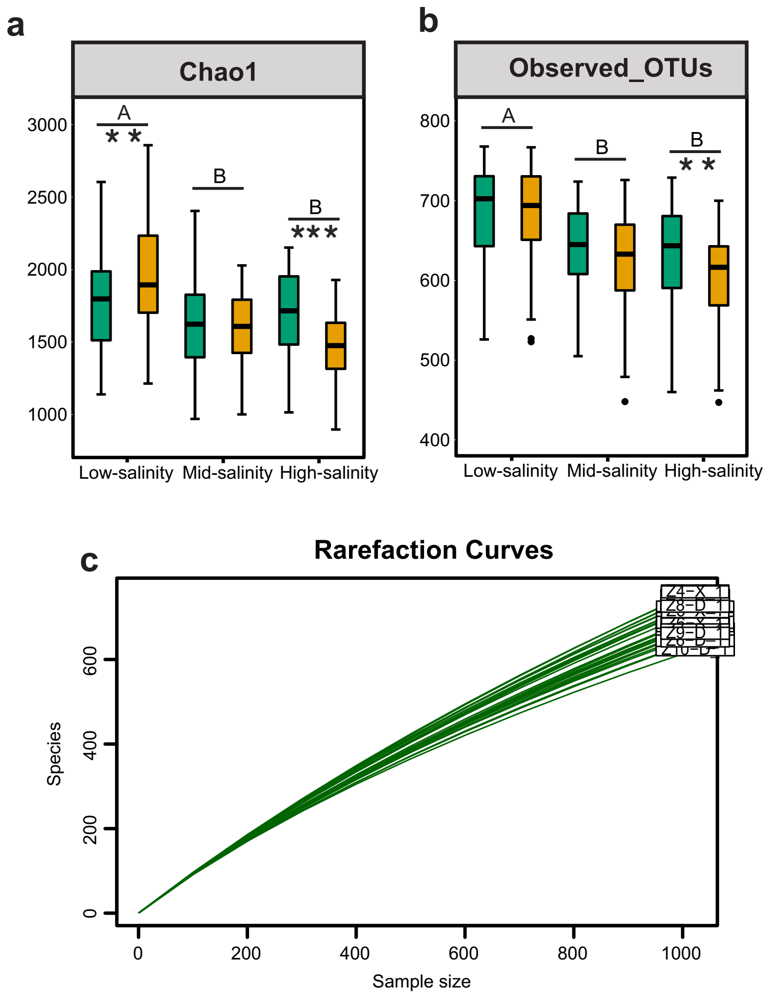

Supplementary Figure 4 (a, b) Boxplots showing alpha-diversity metrics (Chao1 and observed OTUs) of sediment microbial communities across low-, mid-, and high-salinity groups in two seasons. Kruskal-Wallis tests were used for multiple-group comparisons, and Wilcoxon tests for pairwise seasonal comparisons. Different letters indicate significant differences among salinity groups (P < 0.05). Asterisks denote significance levels: * $P < 0.05$, ** $P < 0.01$, *** $P < 0.001$. (c) Rarefaction curves based on the universal single-copy marker gene *rplB*, showing OTU recovery across samples.

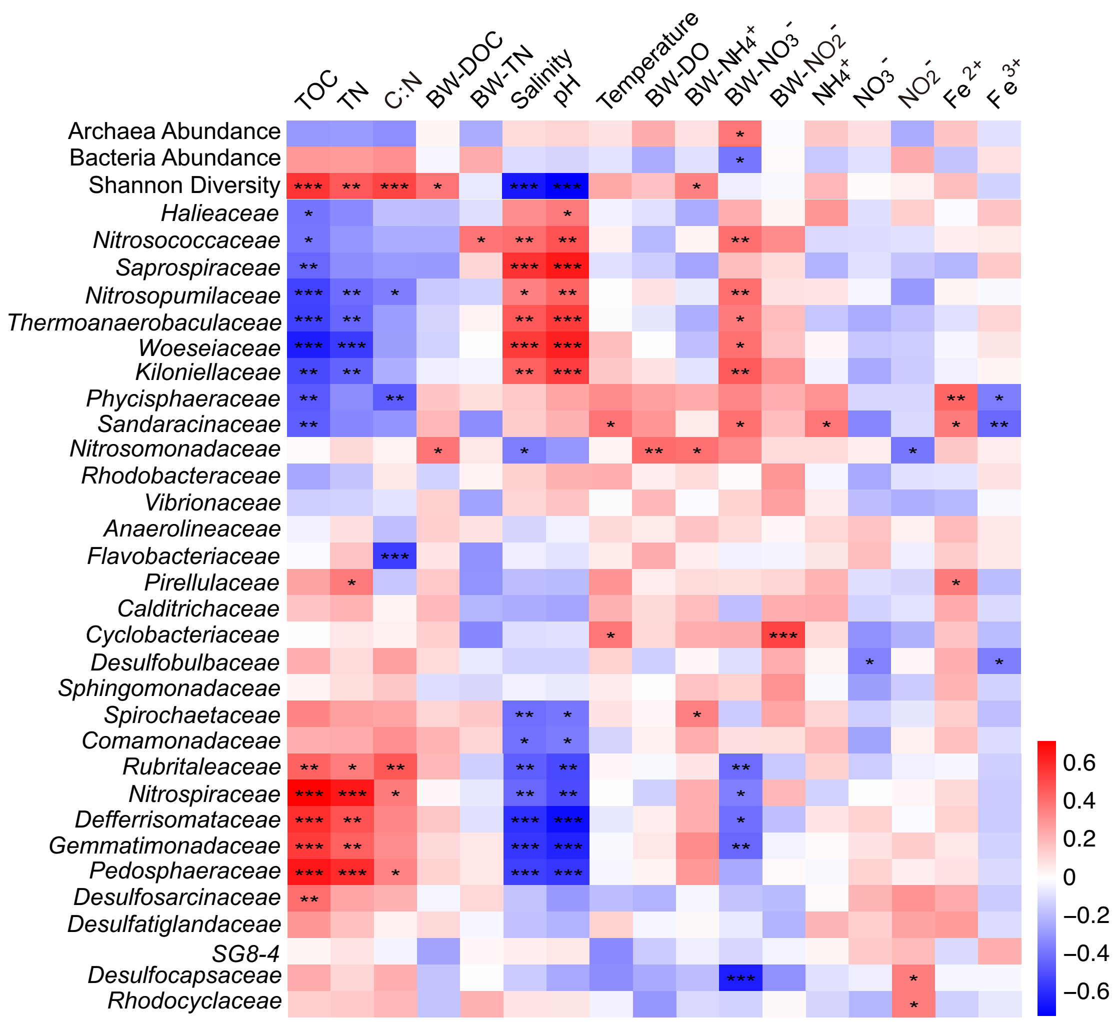

Supplementary Figure 5 Correlation analysis between environmental factors and microbial communities based on 16S mitags in Zhenhai Bay sediments. Red and blue indicate positive and negative correlation respectively, and the asterisk indicates the P-value of the correlation. The P-value test is carried out at α =0.05, *** means $P < 0.01$, ** means $P < 0.05$, * means $P < 0.1$. Detailed statistics for environmental factors of each sample can be found in Supplementary Table 2.

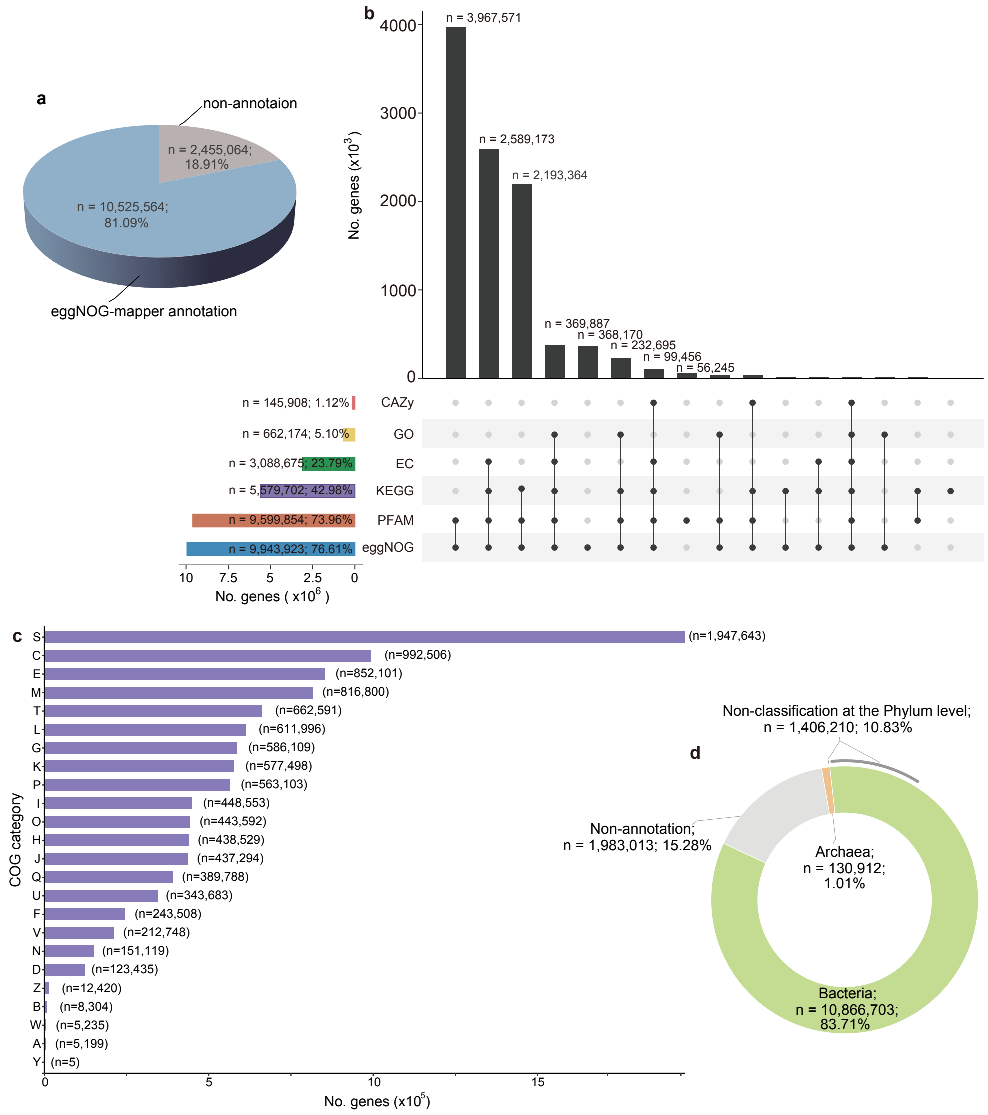

Supplementary Figure 6 Functional and taxonomic information of the non-redundant gene catalog of the Zhenhai Bay sediments. (a) The proportion of annotated versus non-annotated genes. 'Non-annotation' denotes genes that lack annotations in at least one of the following databases: eggNOG, Pfam, KEGG, EC, GO, and CAZy. (b) Distribution of genes with functional annotations across six different databases. The vertical bars depict the number of genes specific to each functional database (colored) or shared among multiple databases (black). The horizontal bars in the lower panel show the total count of genes annotated in each database. (c) Bar chart displaying functional annotations at the COG (Clusters of Orthologous Groups) category level. (d) Taxonomic classification for the non-redundant gene catalog, highlighting the proportion of bacterial genes.

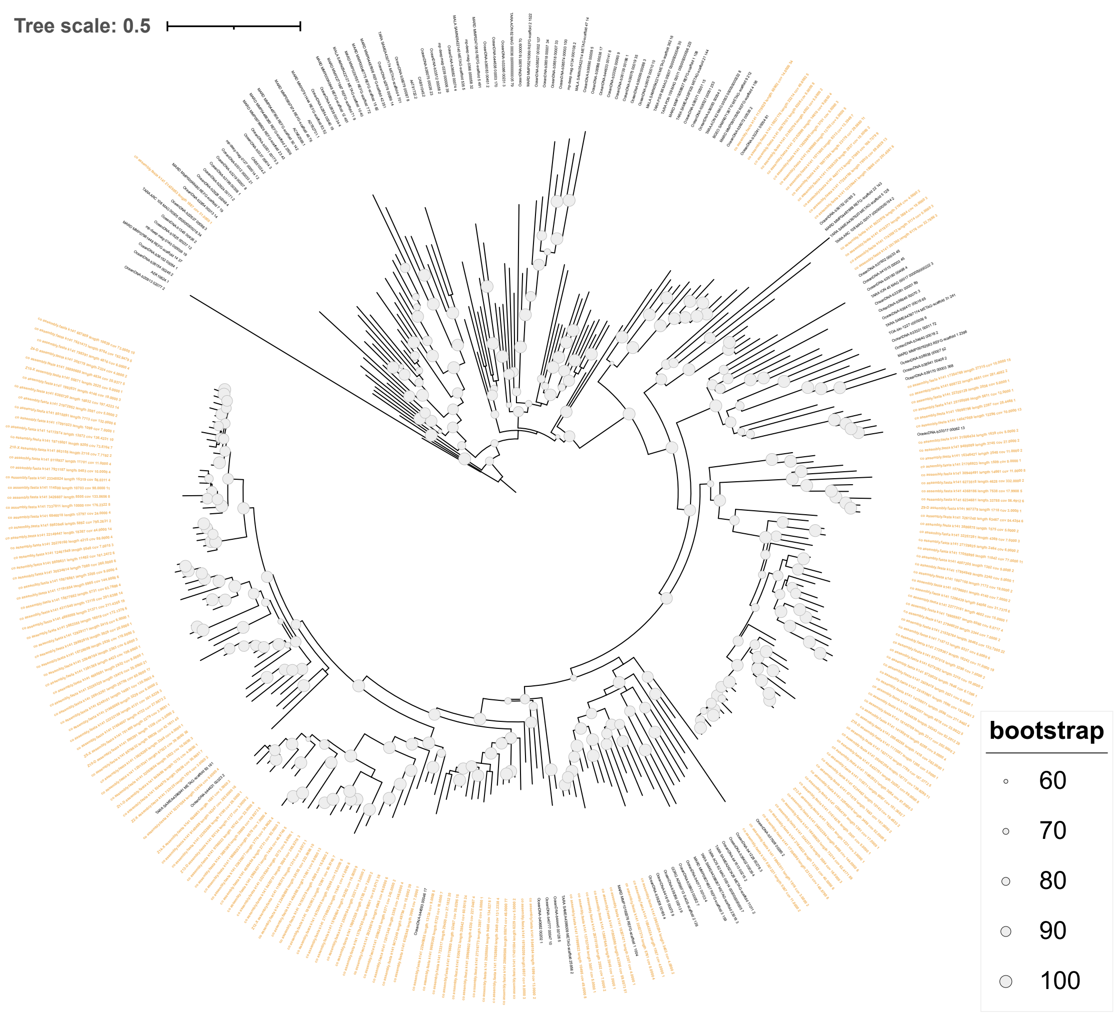

Supplementary Figure 7 Maximum-likelihood phylogenetic tree of AlkB protein sequences recovered from metagenomic datasets and reference sequences. Sequences from Zhenhai Bay sediments are color-coded. Scale bars indicate the mean number of amino acid substitutions per site.

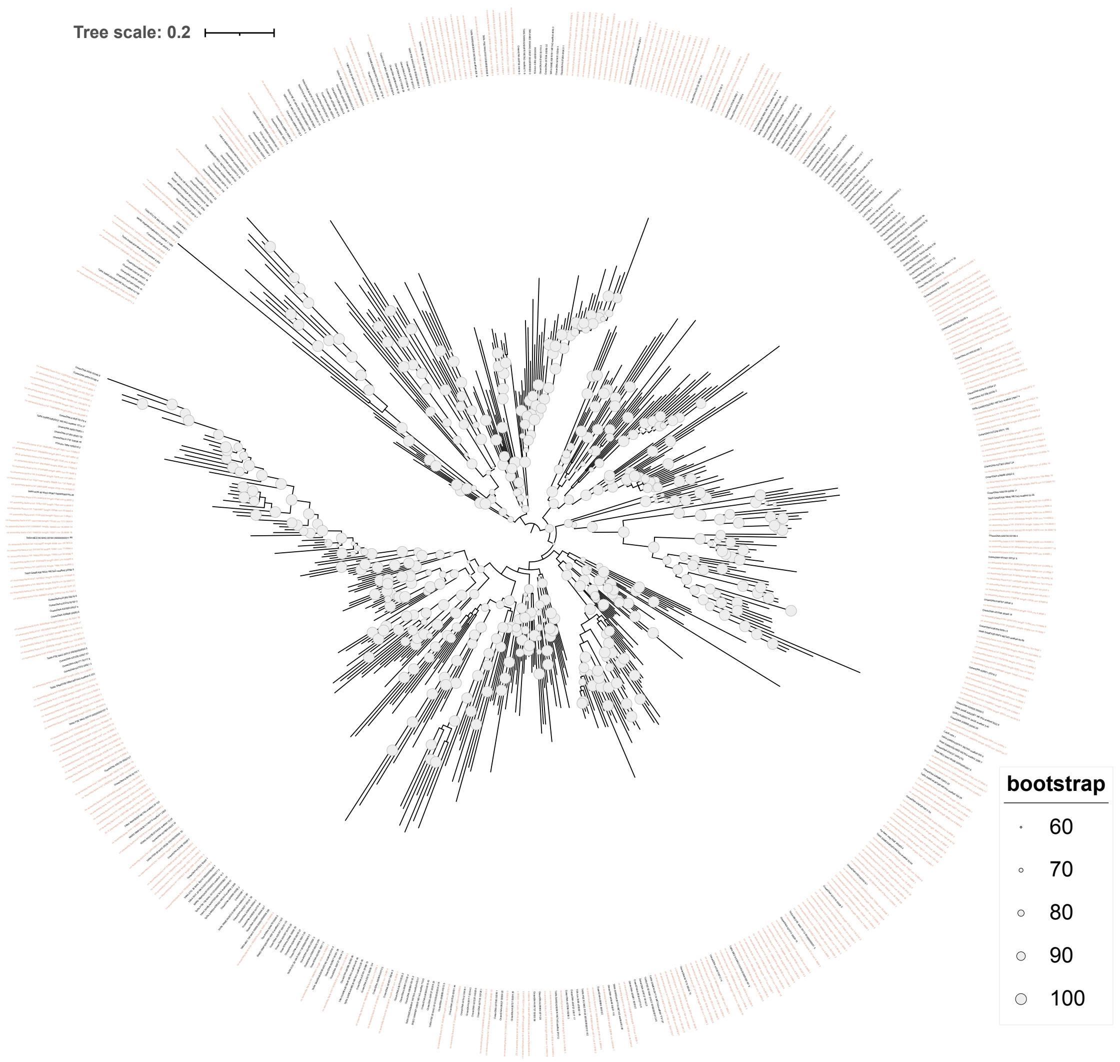

Supplementary Figure 8 Maximum-likelihood phylogenetic tree of CYP153 protein sequences recovered from metagenomic datasets and reference sequences. Sequences from Zhenhai Bay sediments are color-coded. Scale bars indicate the mean number of amino acid substitutions per site.

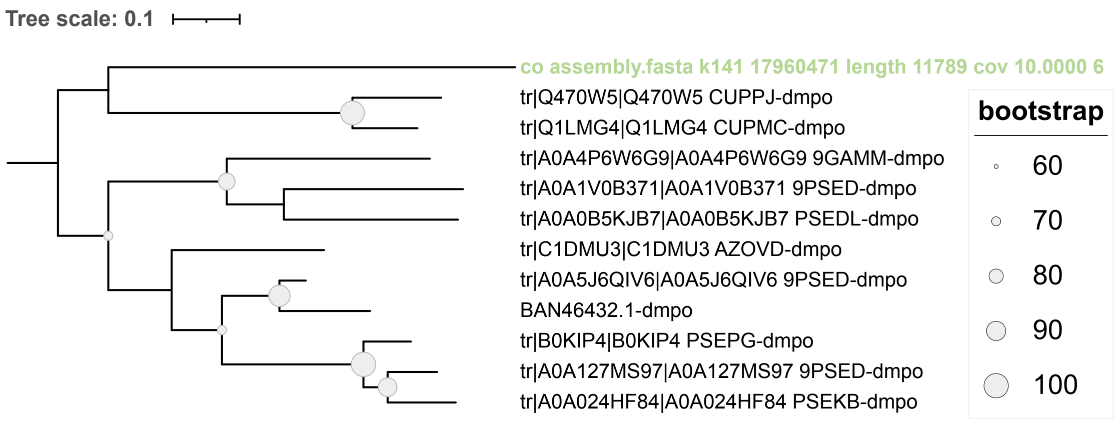

Supplementary Figure 9 Maximum-likelihood phylogenetic tree of DmpO protein sequences recovered from metagenomic datasets and reference sequences. Sequences from Zhenhai Bay sediments are color-coded. Scale bars indicate the mean number of amino acid substitutions per site.

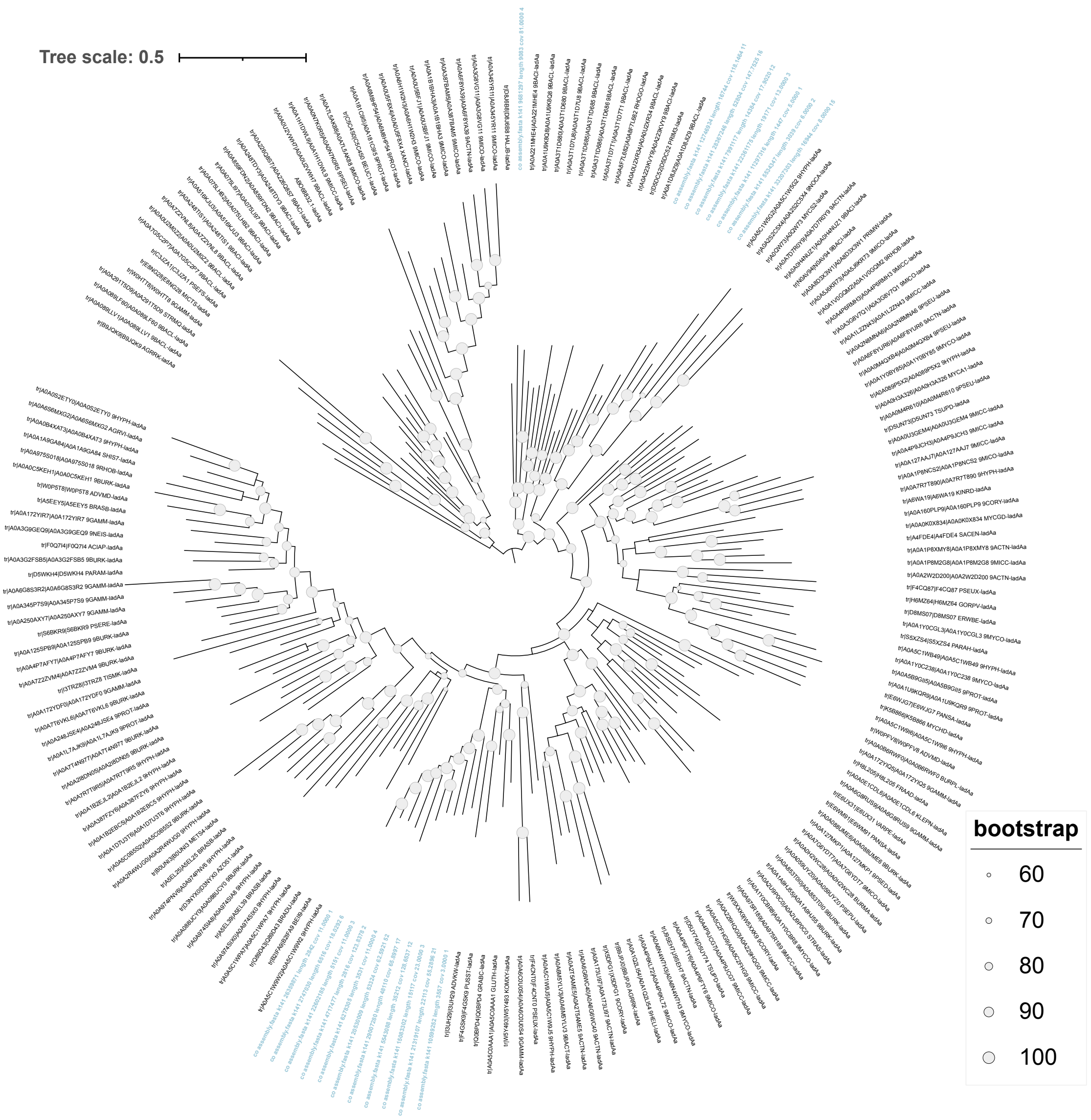

Supplementary Figure 10 Maximum-likelihood phylogenetic tree of LadA_alpha protein sequences recovered from metagenomic datasets and reference sequences. Sequences from Zhenhai Bay sediments are color-coded. Scale bars indicate the mean number of amino acid substitutions per site.

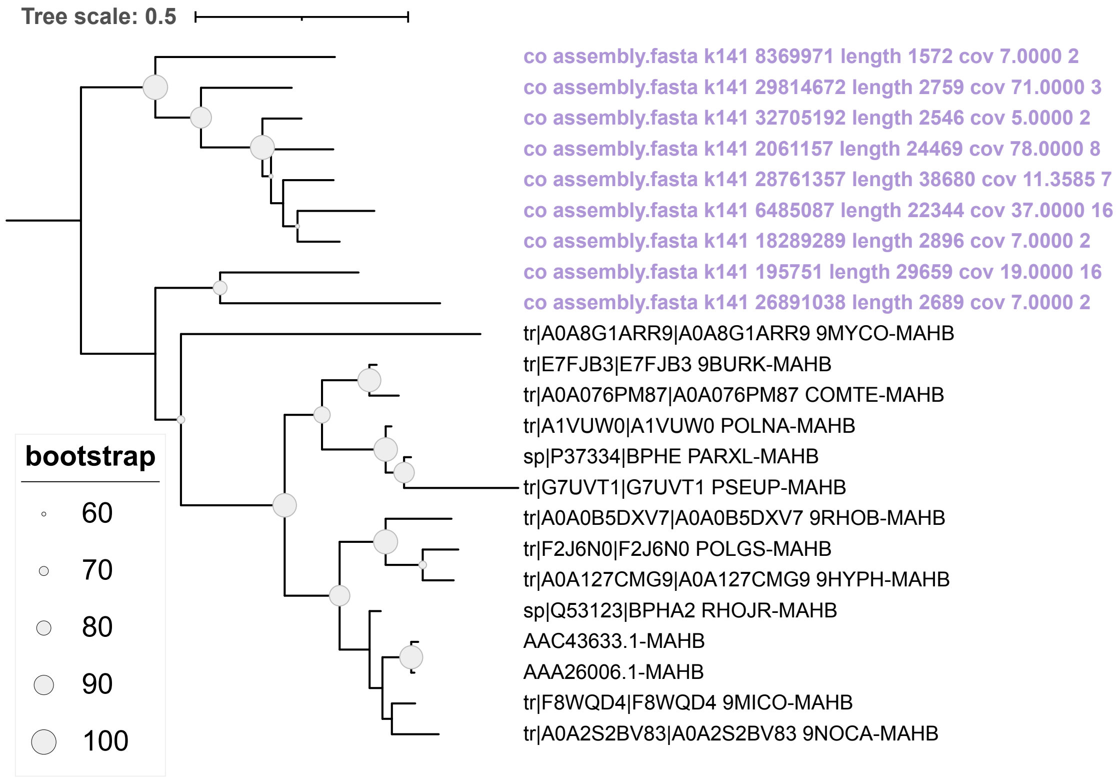

Supplementary Figure 11 Maximum-likelihood phylogenetic tree of MAH_beta protein sequences recovered from metagenomic datasets and reference sequences. Sequences from Zhenhai Bay sediments are color-coded. Scale bars indicate the mean number of amino acid substitutions per site.

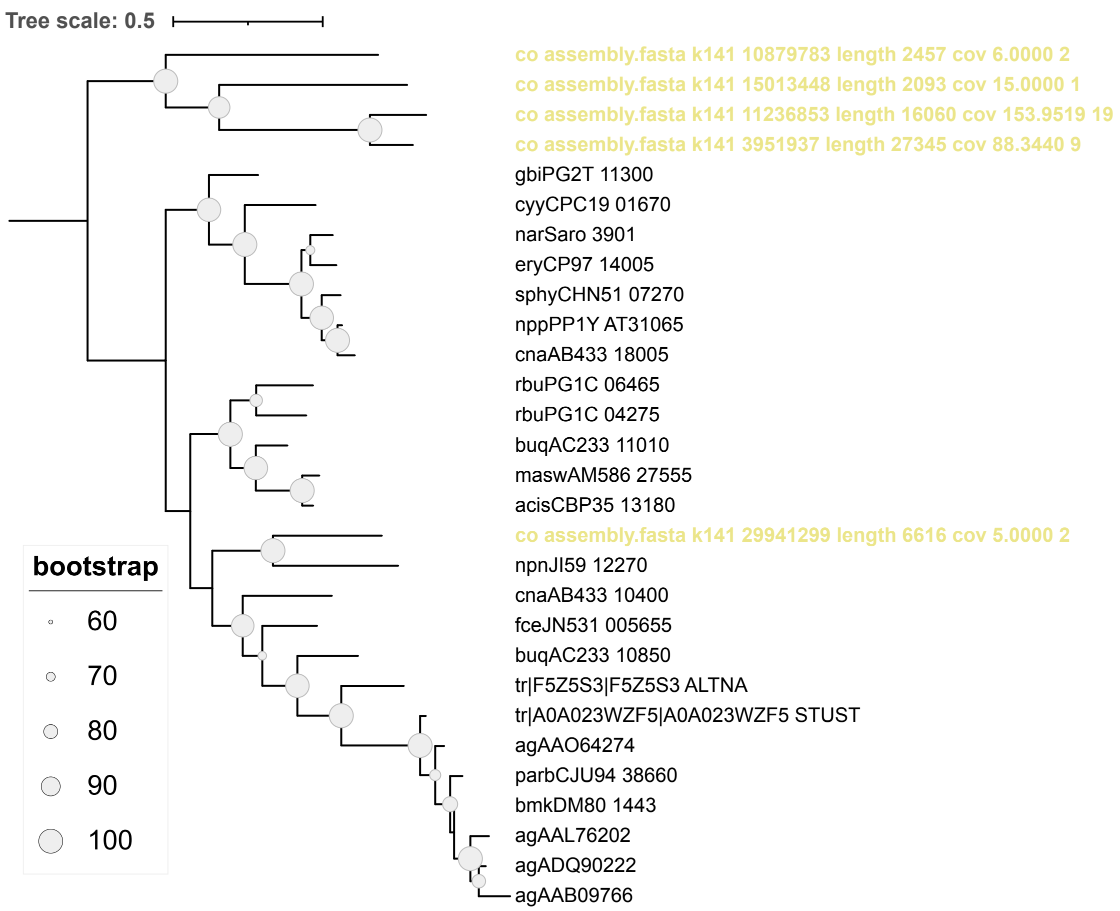

Supplementary Figure 12 Maximum-likelihood phylogenetic tree of NdoB protein sequences recovered from metagenomic datasets and reference sequences. Sequences from Zhenhai Bay sediments are color-coded. Scale bars indicate the mean number of amino acid substitutions per site.

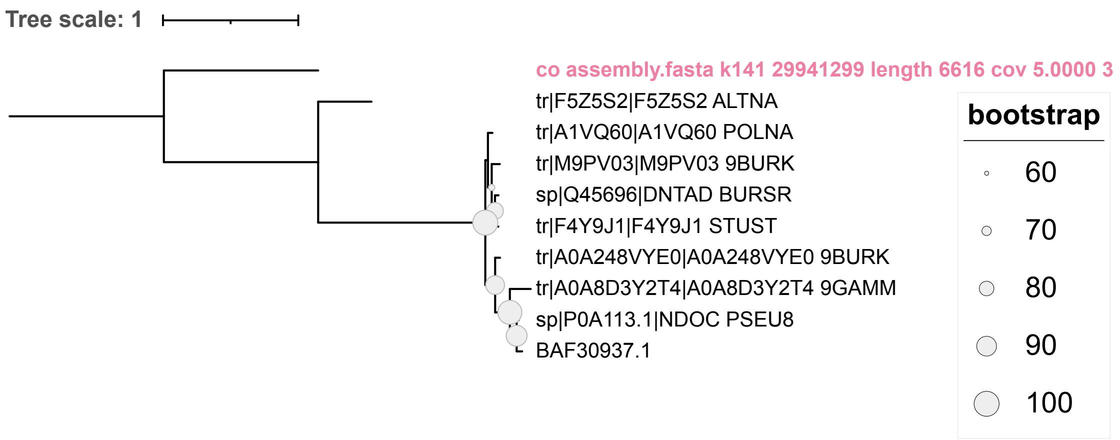

Supplementary Figure 13 Maximum-likelihood phylogenetic tree of NdoC protein sequences recovered from metagenomic datasets and reference sequences. Sequences from Zhenhai Bay sediments are color-coded. Scale bars indicate the mean number of amino acid substitutions per site.

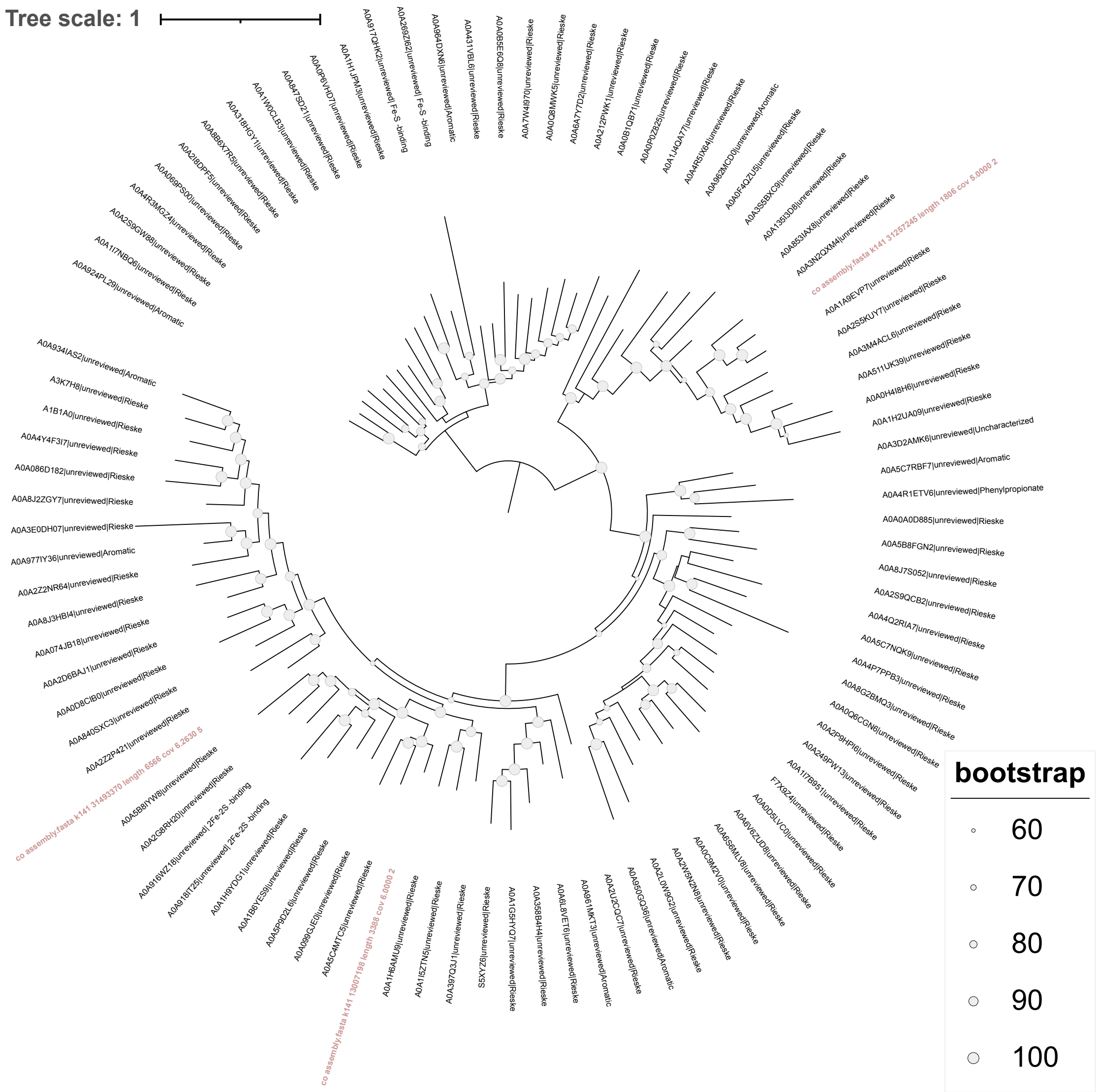

Supplementary Figure 14 Maximum-likelihood phylogenetic tree of non-NdoB type protein sequences recovered from metagenomic datasets and reference sequences. Sequences from Zhenhai Bay sediments are color-coded. Scale bars indicate the mean number of amino acid substitutions per site.

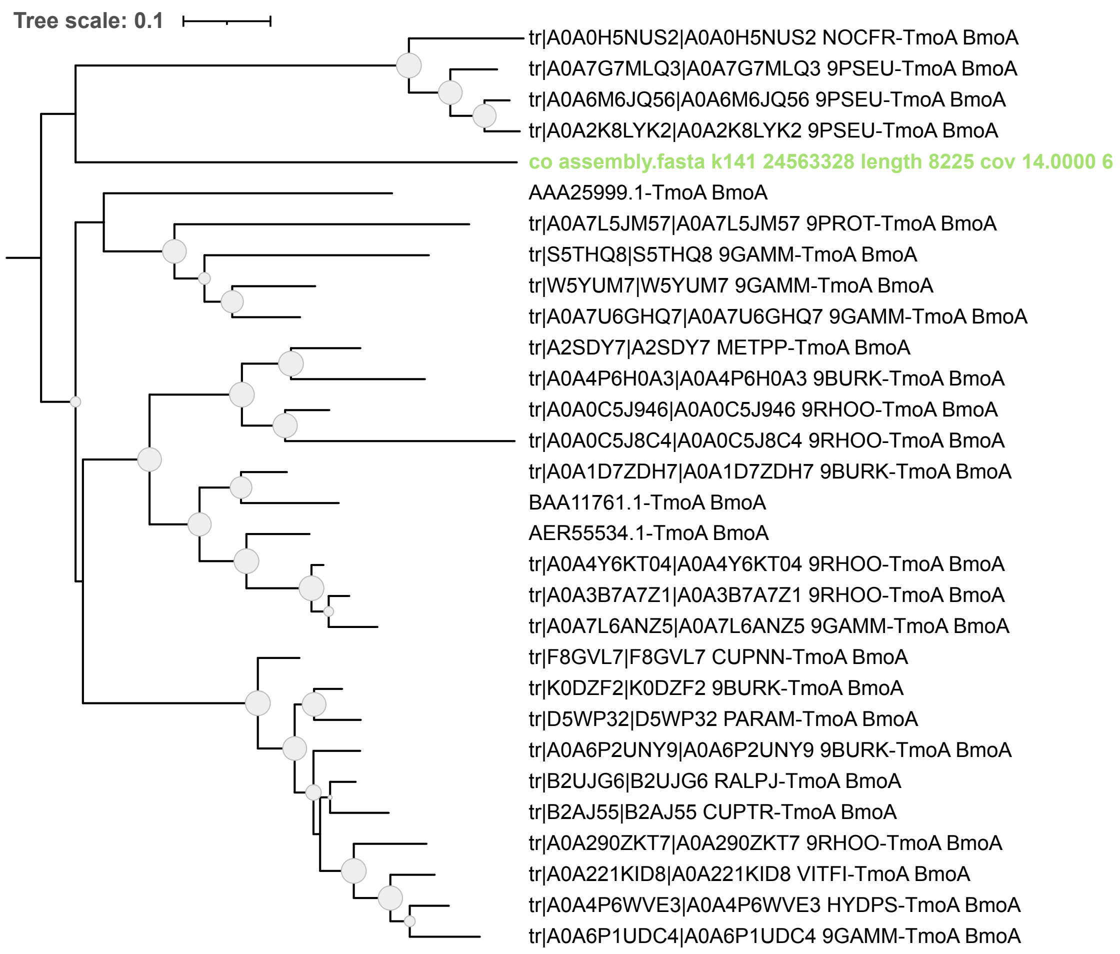

Supplementary Figure 15 Maximum-likelihood phylogenetic tree of TmoA/BmoA protein sequences recovered from metagenomic datasets and reference sequences. Sequences from Zhenhai Bay sediments are color-coded. Scale bars indicate the mean number of amino acid substitutions per site.

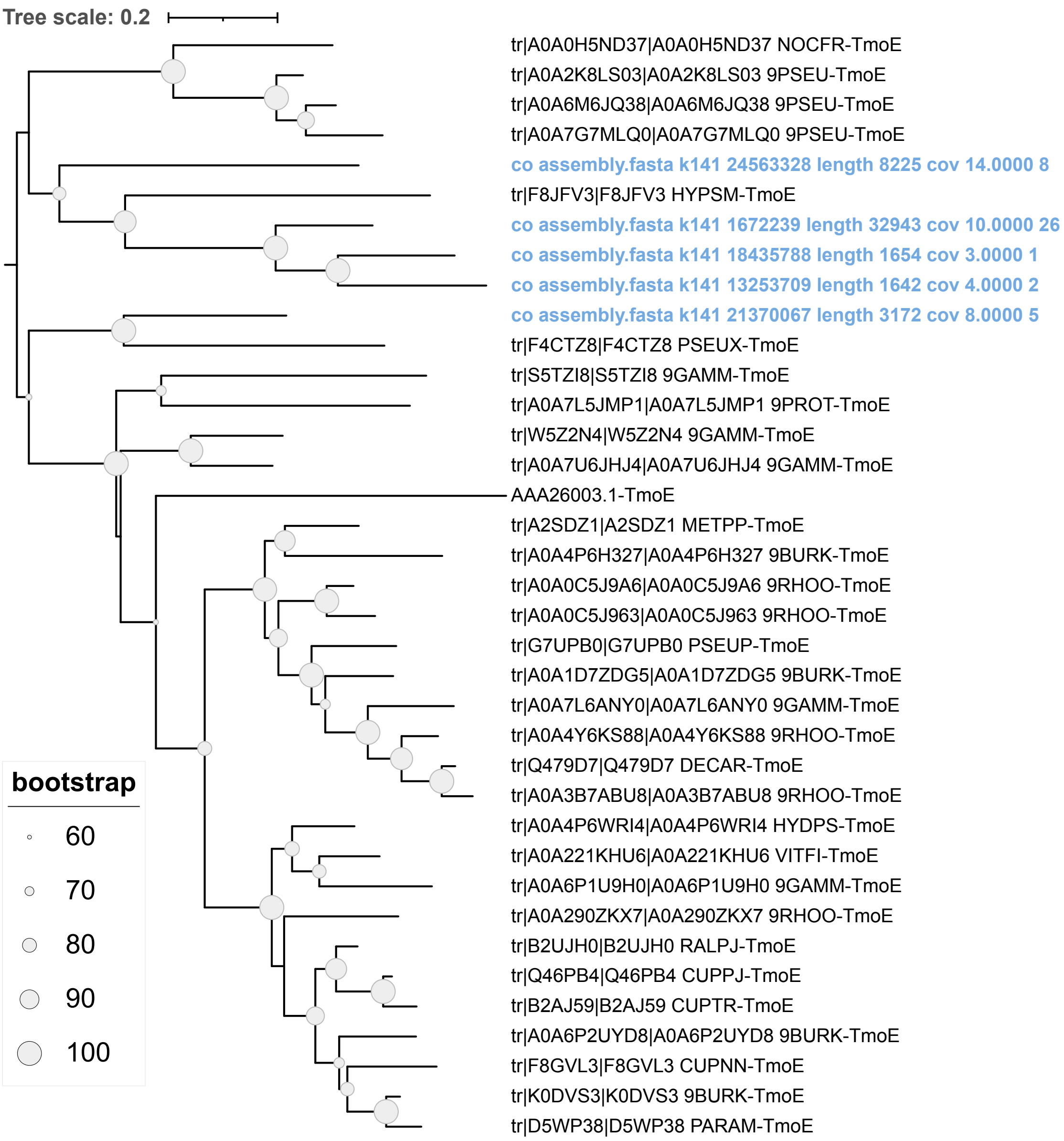

Supplementary Figure 16 Maximum-likelihood phylogenetic tree of TmoE protein sequences recovered from metagenomic datasets and reference sequences. Sequences from Zhenhai Bay sediments are color-coded. Scale bars indicate the mean number of amino acid substitutions per site.

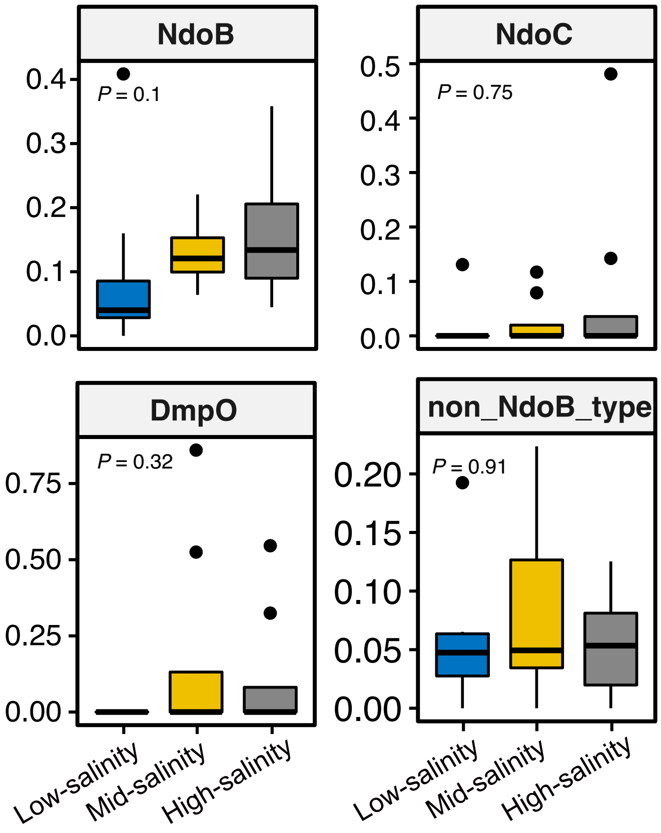

Supplementary Figure 17 Gene abundances (GPM, genes per million) of four types of aerobic hydrocarbon-degrading genes across three salinity groups. Detailed data for hydrocarbon-degrading genes are provided in Supplementary Table 5.

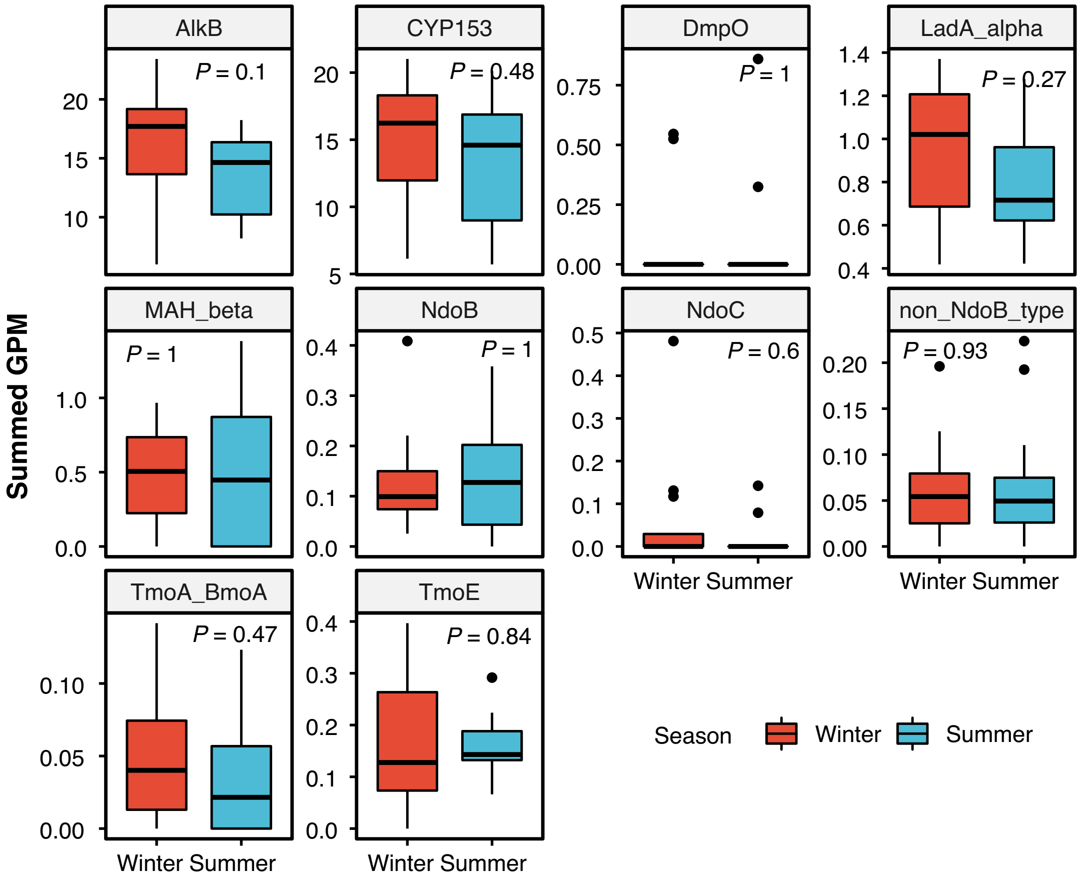

Supplementary Figure 18 Gene abundances (GPM, genes per million) of aerobic hydrocarbon-degrading genes between two season groups. Detailed data for hydrocarbon-degrading genes are provided in Supplementary Table 5.

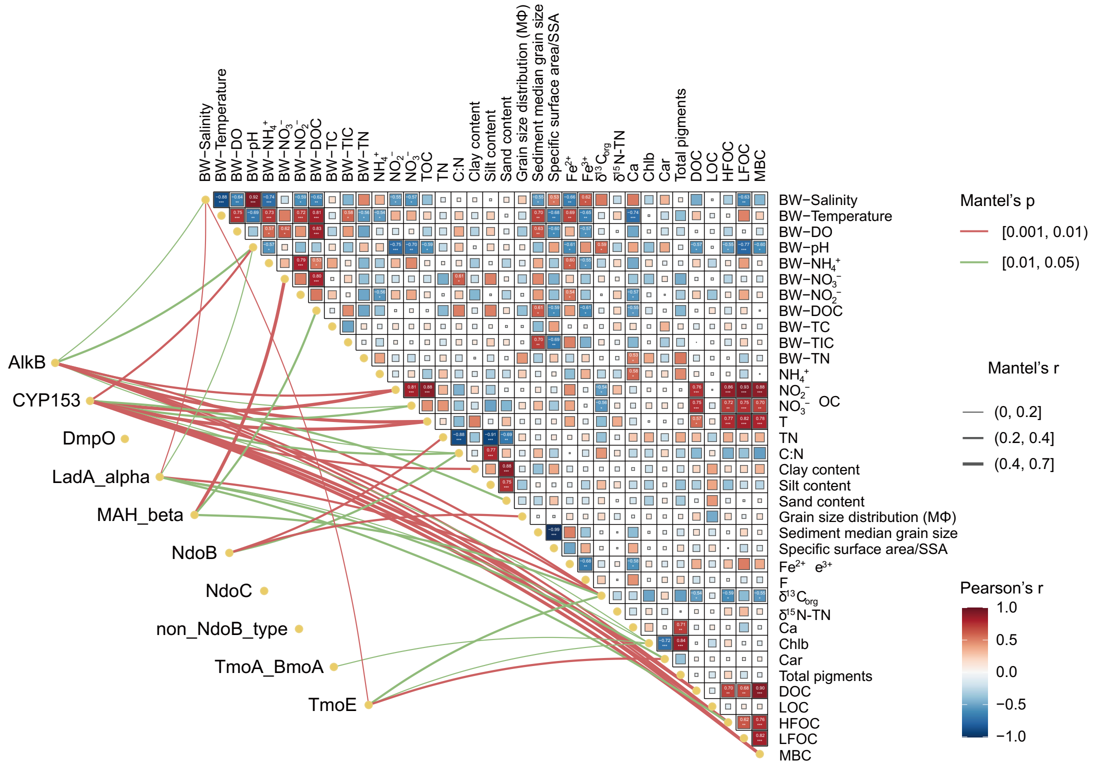

Supplementary Figure 19 Pairwise correlations among environmental variables (heatmap) and their Mantel-test associations with hydrocarbon-degrading genes. Color scale indicates Pearson's r (red = positive, blue = negative). Line width represents Mantel's r; color denotes significance (red < 0.01, green < 0.05, gray > 0.05). Detailed correlation data are provided in Supplementary Table 7.

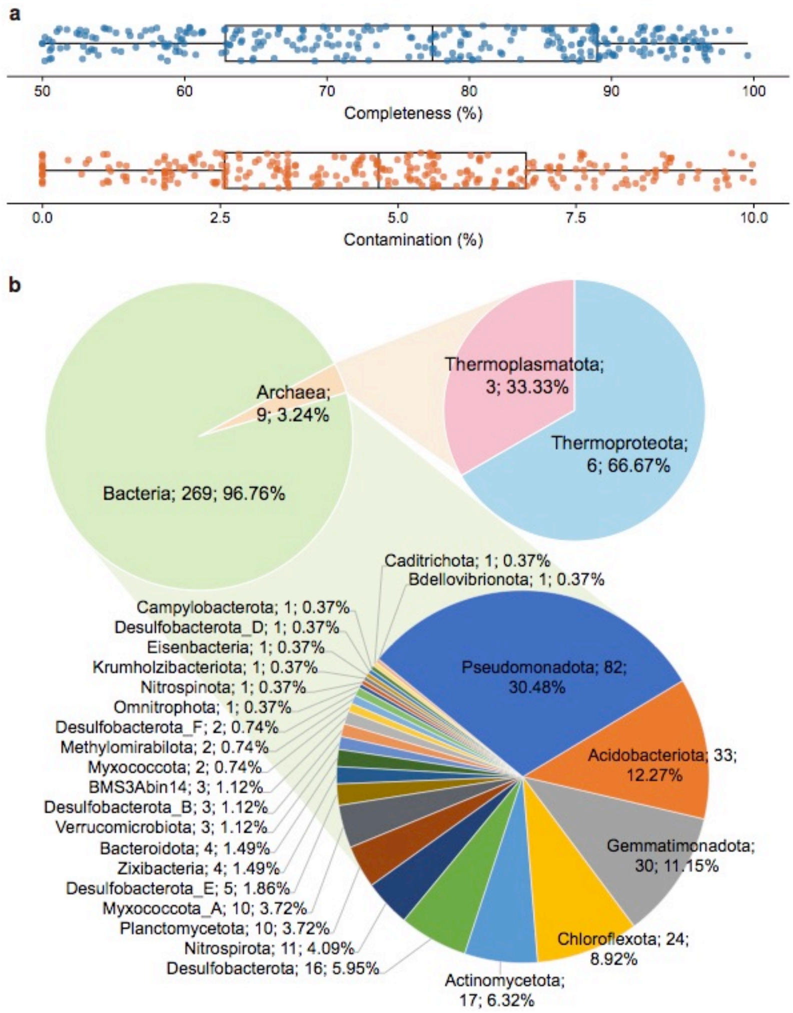

Supplementary Figure 20 Genome statistics for 278 representative MAGs from the Zhenhai Bay sediments. (a) The completeness and contamination of representative MAGs. (b) Taxonomic classification of the species-level representative MAGs at phylum level. Detailed statistics for species-level representative MAGs can be found in Supplementary Table 8.

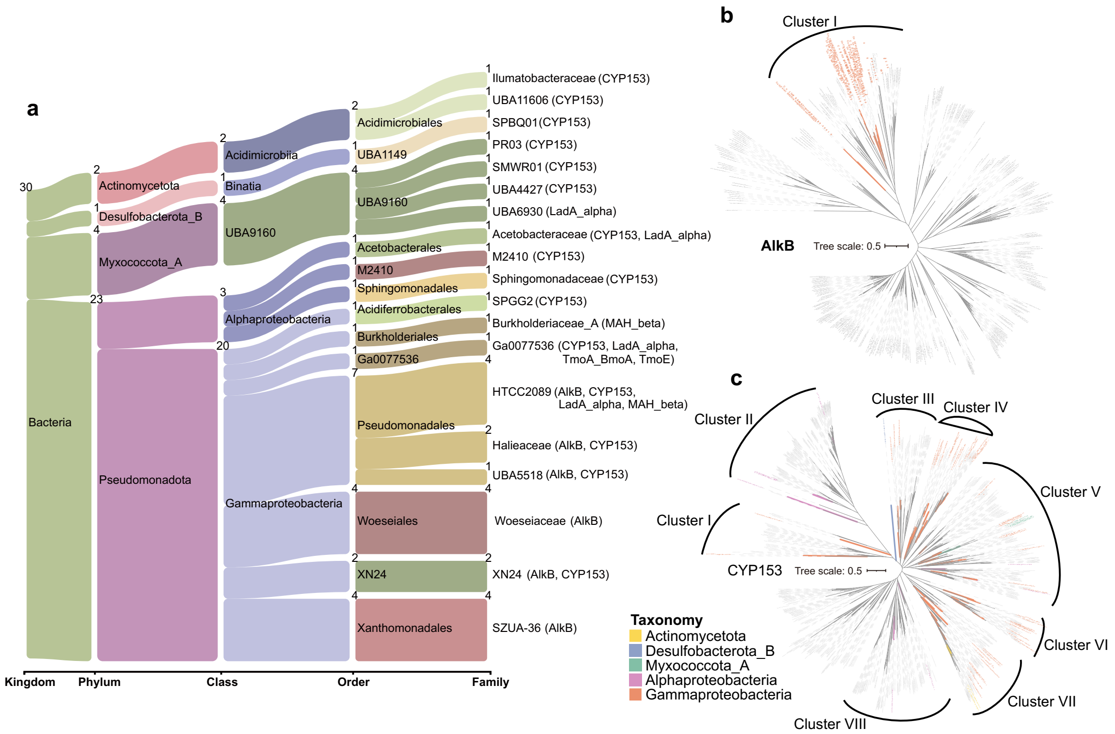

Supplementary Figure 21 Overview of hydrocarbon-degrading bacteria in the Zhenhai Bay microbial community. (a) Sankey diagram showing the taxonomic affiliations of hydrocarbon-degrading MAGs across hierarchical levels based on GTDB taxonomy. (b, c) Maximum-likelihood phylogenetic trees of AlkB (b) and CYP153 (c) protein sequences. Sequences from the same taxa are color-coded; scale bars indicate mean substitutions per site.

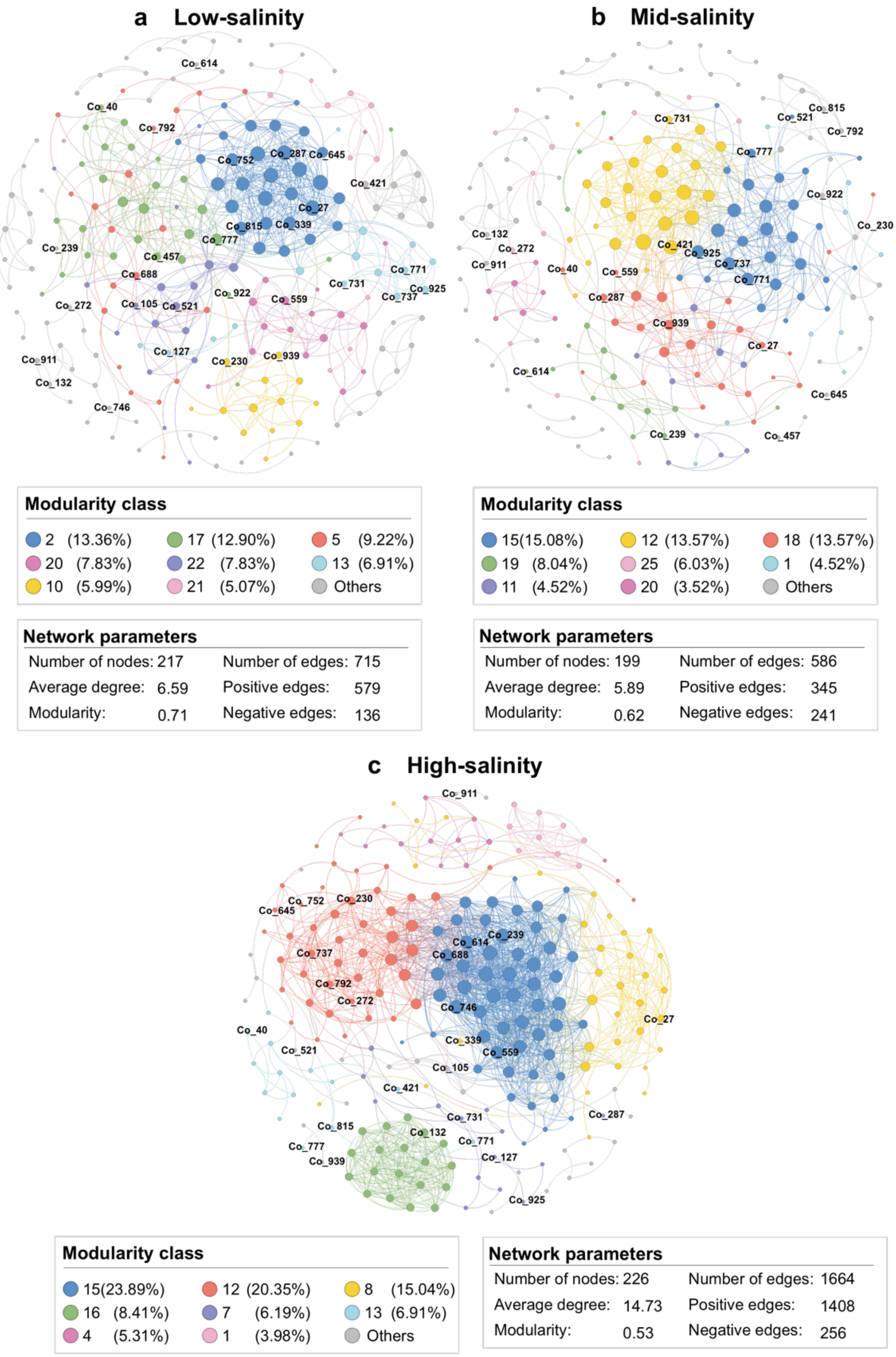

Supplementary Figure 22 Co-occurrence networks of microbial community in Zhenhai Bay sediments across three salinity groups. The top 8 modules with the largest number of nodes are shown in different colors, and smaller modules are shown in grey. The hydrocarbon-degrading MAGs in the networks are highlighted. Detailed statistics for co-occurrence network can be found in Supplementary Table 10.

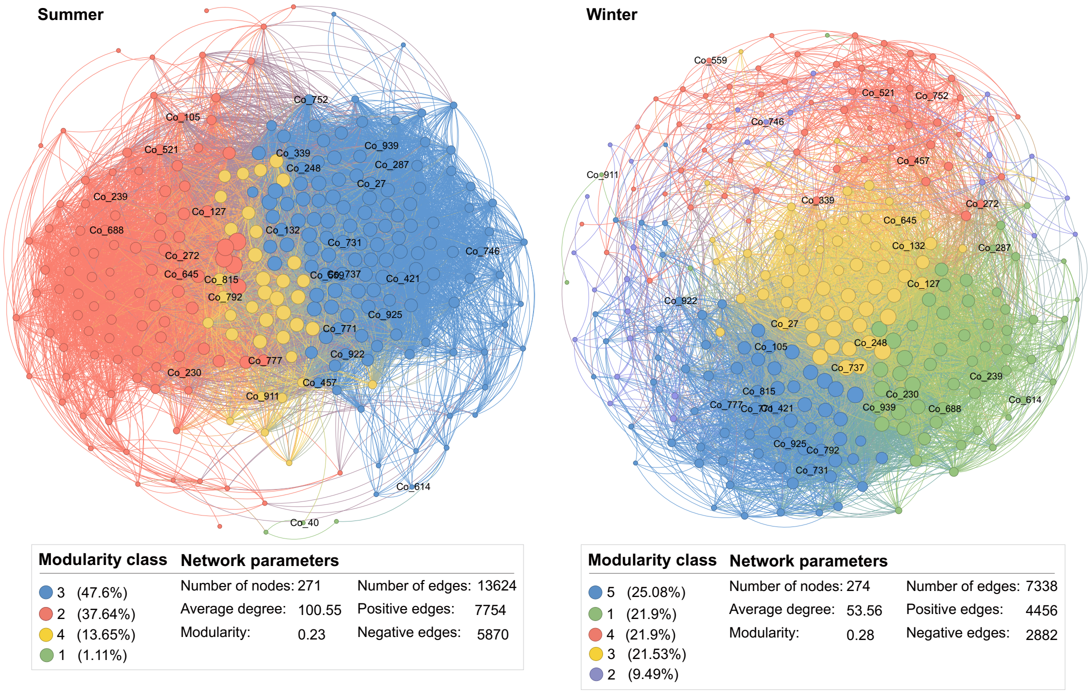

Supplementary Figure 23 Co-occurrence networks of microbial community in Zhenhai Bay sediments in summer and winter. The hydrocarbon-degrading MAGs in the network are highlighted. Detailed statistics for co-occurrence network can be found in Supplementary Table 10.

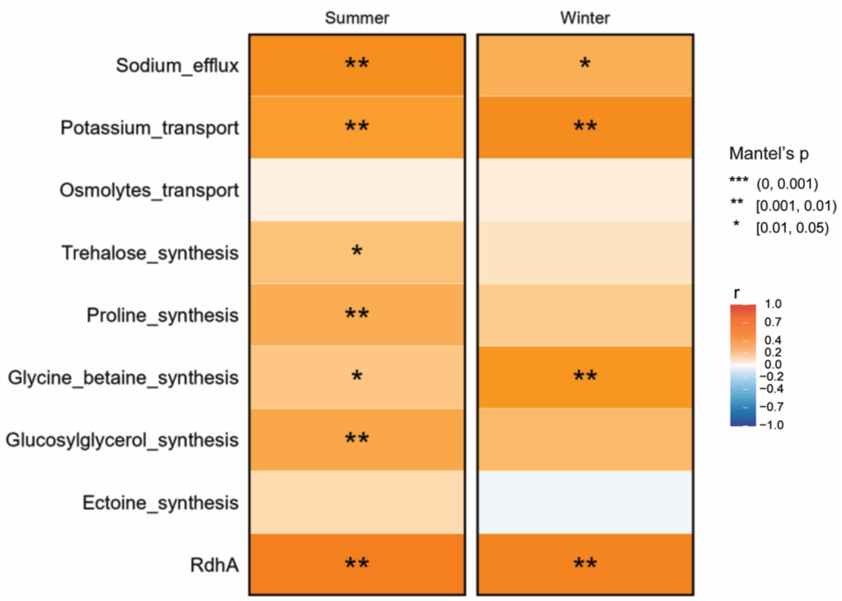

Supplementary Figure 24 Correlation analysis between hydrocarbon-degrading genes and halotolerant genes between two seasons. Red and blue indicate positive and negative correlation respectively, and the asterisk indicates the P-value of the correlation. The P-value test is carried out at α =0.05, *** means $P < 0.001$, ** means $P < 0.01$, * means $P < 0.5$. Detailed data for halotolerant genes can be found in Supplementary Table 13.

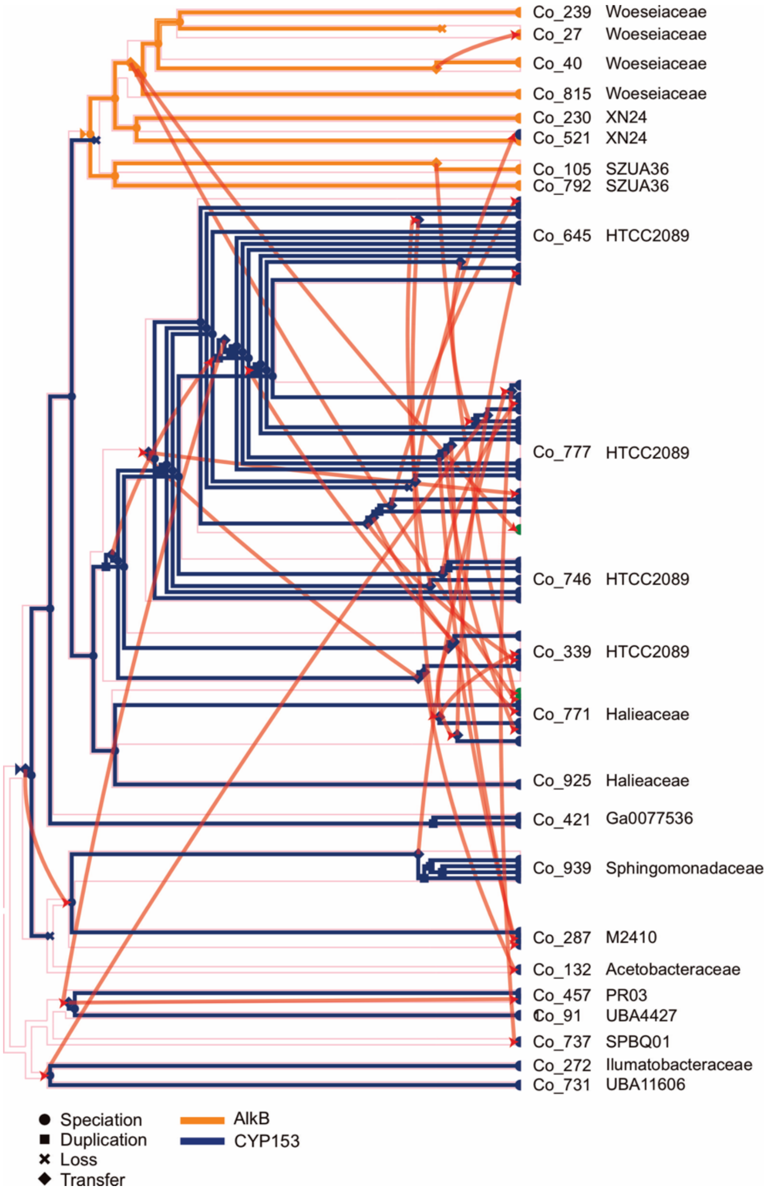

Supplementary Figure 25 Gene-species reconciliation of alkane monooxygenases AlkB (orange) and CYP153 (blue) across hydrocarbon-degrading bacteria. Speciation events are shown as circles, duplications as squares, gene losses as crosses, and horizontal transfers as diamonds. Red lines indicate transfer connections.

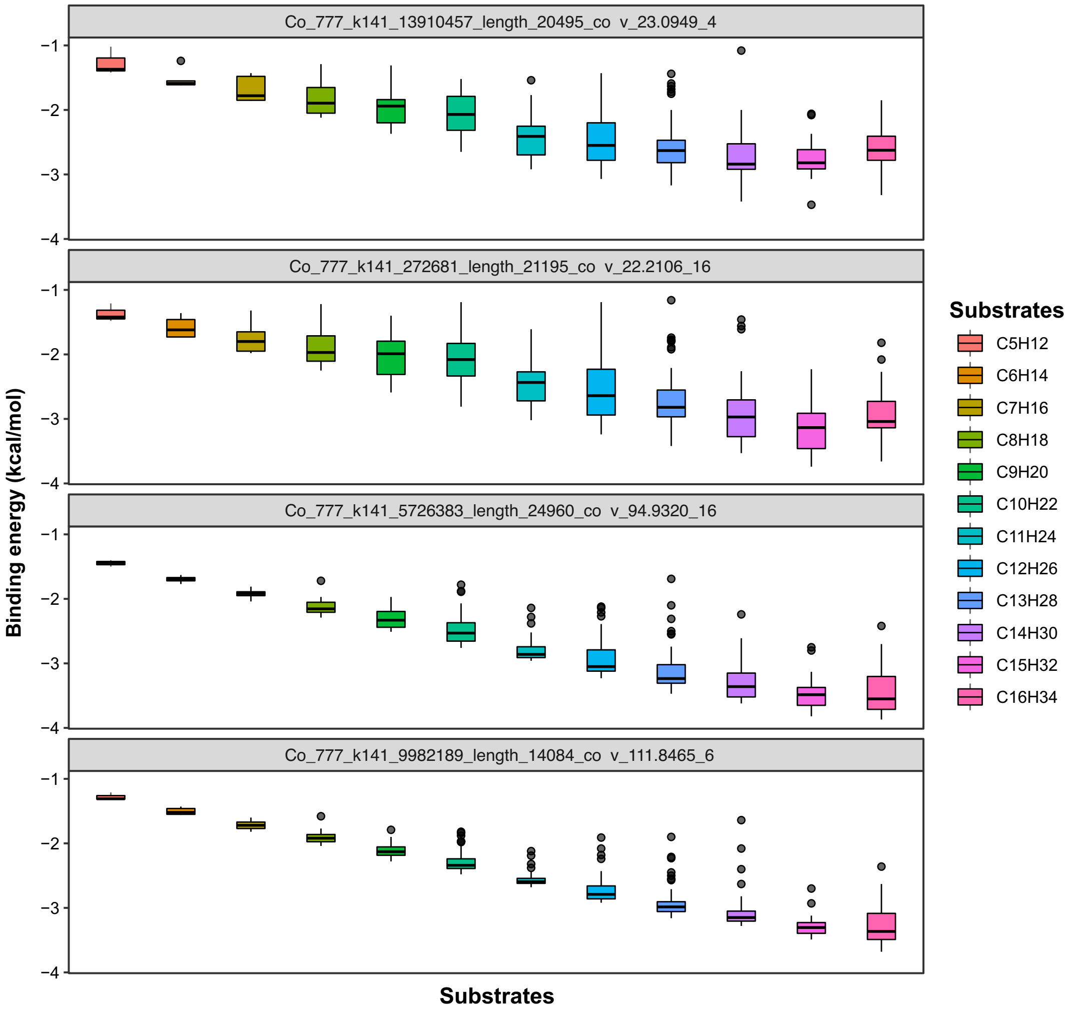

Supplementary Figure 26 The boxplots show the binding energies of CYP153s within Co_777 genome for C5-C16 substrates. Detailed data for molecular docking can be found in Supplementary Table 15.
